# Discovery of BPR1K871, a quinazoline based, multi-kinase inhibitor for the treatment of AML and solid tumors: Rational design, synthesis, *in vitro* and *in vivo* evaluation

**DOI:** 10.18632/oncotarget.13369

**Published:** 2016-11-15

**Authors:** Yung Chang Hsu, Mohane Selvaraj Coumar, Wen-Chieh Wang, Hui-Yi Shiao, Yi-Yu Ke, Wen-Hsing Lin, Ching-Chuan Kuo, Chun-Wei Chang, Fu-Ming Kuo, Pei-Yi Chen, Sing-Yi Wang, An-Siou Li, Chun-Hwa Chen, Po-Chu Kuo, Ching-Ping Chen, Ming-Hsine Wu, Chen-Lung Huang, Kuei-Jung Yen, Yun-I Chang, John T.-A. Hsu, Chiung-Tong Chen, Teng-Kuang Yeh, Jen-Shin Song

**Affiliations:** ^1^ Institute of Biotechnology and Pharmaceutical Research, National Health Research Institutes, Zhunan, Taiwan, ROC; ^2^ Centre for Bioinformatics, School of Life Sciences, Pondicherry University, Kalapet, Puducherry, India; ^3^ Department of Chemistry, National Tsing Hua University, Hsinchu, Taiwan, ROC

**Keywords:** acute myeloid leukemia, aurora kinase, FLT3, quinazoline, multi-kinase inhibitor

## Abstract

The design and synthesis of a quinazoline-based, multi-kinase inhibitor for the treatment of acute myeloid leukemia (AML) and other malignancies is reported. Based on the previously reported furanopyrimidine 3, quinazoline core containing lead 4 was synthesized and found to impart dual FLT3/AURKA inhibition (IC_50_ = 127/5 nM), as well as improved physicochemical properties. A detailed structure-activity relationship study of the lead 4 allowed FLT3 and AURKA inhibition to be finely tuned, resulting in AURKA selective (5 and 7; 100-fold selective over FLT3), FLT3 selective (13; 30-fold selective over AURKA) and dual FLT3/AURKA selective (BPR1K871; IC_50_ = 19/22 nM) agents. BPR1K871 showed potent anti-proliferative activities in MOLM-13 and MV4-11 AML cells (EC_50_ ∼ 5 nM). Moreover, kinase profiling and cell-line profiling revealed BPR1K871 to be a potential multi-kinase inhibitor. Functional studies using western blot and DNA content analysis in MV4-11 and HCT-116 cell lines revealed FLT3 and AURKA/B target modulation inside the cells. *In vivo* efficacy in AML xenograft models (MOLM-13 and MV4-11), as well as in solid tumor models (COLO205 and Mia-PaCa2), led to the selection of BPR1K871 as a preclinical development candidate for anti-cancer therapy. Further detailed studies could help to investigate the full potential of BPR1K871 as a multi-kinase inhibitor.

## INTRODUCTION

Acute myeloid leukemia (AML), an aggressive and frequently fatal hematologic malignancy, is one of the most common types of leukemia in children and adolescents [1]. Chemotherapy with an anthracycline and cytarabine is the most common treatment for AML; but is not always well tolerated [2]. Moreover, the 5-year survival rate of patients with AML (24%) is much lower than the average with all cancers (68%) [1]. Accordingly, newer, more effective, less toxic treatments for AML are urgently required.

In AML patients, many gene mutations have been identified during the past 40 years, such as NPM1, PLK1, MLL, FLT3, and JAK2 [3]. The relevant protein products? have been evaluated as potential therapeutic targets for treating AML. FMS-like receptor tyrosine kinase-3 (FLT3), encoded by the FLT3 gene, belongs to the class III tyrosine kinase receptor family and plays a pivotal role in regulating differentiation, growth, and migration of hematopoietic cells [4]. In AML, an internal tandem duplication mutation of FLT3 (FLT3/ITD) has been found in about 20–25% of patients and is often associated with poor prognosis and high risk for relapse [5]. Although FLT3-selective inhibition is proven to be an effective AML therapeutic strategy, clinical benefits are generally limited due to the emergence of resistance [6]. Several studies now demonstrate that targeting both FLT3-dependant and FLT3-independant pathways is much more beneficial in FLT3/ITD AML patients, and also overcomes acquired resistance to selective FLT3 inhibitors [6–8].

Aurora kinase (AURK) isoforms A, B, and C (AURKA, AURKB, and AURKC), are members of the serine/threonine kinase family and are involved in the regulation of various stages of mitosis [9]. Several studies demonstrate that Aurora kinases, particularly AURKA, are over-expressed in various tumors, highly associated with the abnormal growth of cancer cells [9], and may serve as an additional biomarker for AML [7]. A biomarker, or biological marker, is a measurable parameter which could be used to determine the severity of a disease condition as well as the effectiveness of a treatment. As, AURK inhibition levels are corroborated with the efficacy of several inhibitors in preclinical AML models, AURK is considered as a useful biomarker in addition to FLT3 for AML treatment. Therefore, dual FLT3/AURK inhibitors are emerging as an exciting new generation of AML therapeutics.

Many research groups have reported small molecule dual FLT3 and AURK inhibitors that could be beneficial for the treatment of AML; for example, barasertib developed by AstraZeneca and currently in phase II/III clinical trials [10]; CCT241736 developed by the Cancer Research UK Cancer Therapeutics Unit and currently in the preclinical stage [11]; an indolinone derivative from Chern et al. [12]; and a pyrrolopyrimidine derivative from our laboratory [13].

Herein, we report the design and synthesis of a quinazoline-based multi-kinase inhibitor for the treatment of AML and other malignancies. Structure-activity relationship (SAR) exploration in this series led to the identification of BPR1K871 with potent dual enzymatic (AURKA IC_50_ = 22 nM; AURKB = 13 nM; FLT3 IC_50_ = 19 nM) and cellular activities in AML cell lines (MOLM-13 and MV4-11; EC_50_ ∼ 5 nM). Kinase profiling of BPR1K871 using the KINOMEScan technology revealed that 77 therapeutically important kinases out of 395 non-mutant kinases were inhibited 65% at 1000 nM. Based on the multi-kinase inhibition potential, BPR1K871 was tested in a panel of 15 cancer cell lines to investigate the full anti-proliferative potential. In addition to AML cell lines, BPR1K871 inhibited COLO205 and Mia-PaCa2 cell lines potently (EC_50_ < 100 nM). More importantly, BPR1K871 exhibited excellent *in vivo* efficacy not only in leukemia MOLM-13 and MV4-11 but also in colorectal COLO205 and pancreatic Mia-PaCa2 xenograft models (3–20 mg/ kg, iv) without significant toxicity. *In vitro* and *in vivo* experiments indicated that BPR1K871 is a multi-kinase inhibitor which may provide therapeutic benefit over existing treatment and is currently selected as a potential lead candidate for further preclinical investigations.

## RESULTS

### Design of quinazoline-based dual FLT3/AURKA inhibitors

**Figure 1:**
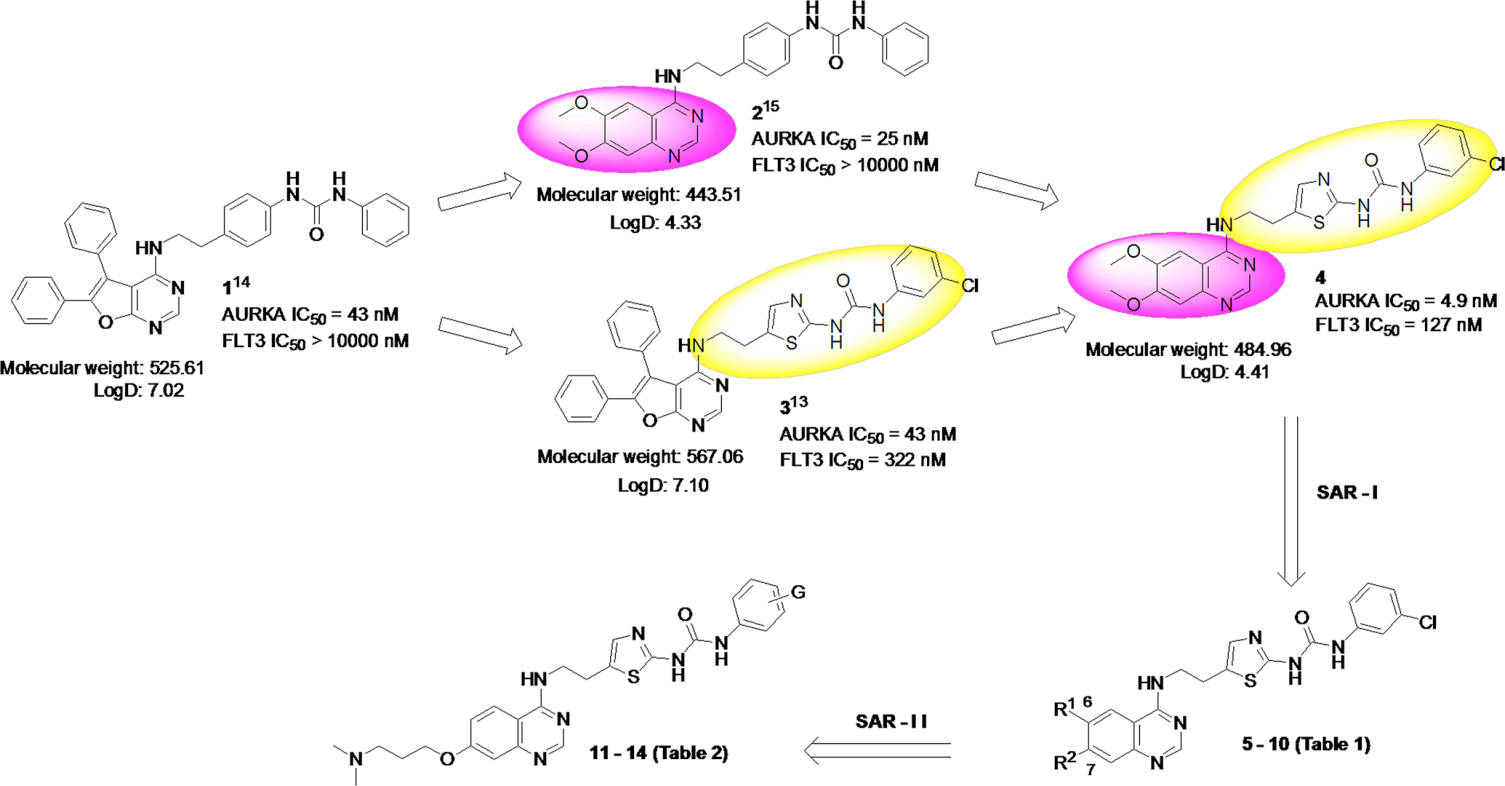
Hybrid design strategy for novel quinazoline-based dual FLT3/AURKA inhibitors

In our effort to develop targeted anti-cancer agents, furanopyrimidine core containing 1 was previously identified as an AURK inhibitor lead (Figure 1) [14]. However, due to lower *in vitro* activity as well as a poor *in vivo* pharmacokinetics profile, attempts were made to modify both the furanopyrimidine core structure as well as the urea side chain of 1. 3D-QSAR based lead optimization efforts led to the identification of quinazoline core based lead 2 with improved *in vitro* activity as well as *in vivo* pharmacokinetics profile [15]. In addition, a variety of urea side chain modifications were explored utilizing a FLT3 homology model developed in-house, to guide the structure-based design efforts. This resulted in the identification of furano-pyrimidine core based 3 with a thiazole containing urea side chain as a dual FLT3/AURKA inhibitor [13]. Lead 2 retained the urea containing side chain of the initial lead 1; while lead 3 retained the furanopyrimidine core of the initial lead 1.

Considering the potential use of a dual FLT3/AURKA inhibitor, here we hybridized 2 and 3 to design quinazoline core based inhibitor 4 with a thiazole containing urea side chain. Particularly, scaffold-hopping from a furanopyrimidine core (3) to quinazoline core (4) was anticipated to improve physicochemical properties such as lipophilicity (LogD_7.4_: 7.10 to 4.41), and also lowered the molecular weight (567 to 485). More importantly, the quinazoline core is considered a privileged structure for the inhibition of ATP-dependent kinases, since 5 out of 30 kinase inhibitors approved by the FDA contain the quinazoline framework [16]. Accordingly, 4 was synthesized and tested for *in vitro* FLT3 and AURKA inhibition as well its ability to inhibit proliferation of AML cell lines (MOLM-13 and MV4-11). Compound 4 showed 5-10 fold improved AURKA inhibition (IC_50_ = 4.9 nM) as compared to 2 and 3 (IC_50_ = 25 and 43 nM), as well as 3-fold improved FLT3 inhibition (IC_50_ = 127 nM) as compared with 3 (IC_50_ = 322 nM). Moreover, 4 inhibited the proliferation of AML cell lines with an EC_50_ ∼ 40 nM. Despite the improved *in vitro* profile, 4 could not be progressed to *in vivo* efficacy evaluation due to poor aqueous solubility (0.452 μg/mL) and dose-limiting toxicity. Hence, we undertook a detailed SAR exploration using 4 as a starting point to identify potent dual FLT3/AURKA inhibitors suitable for preclinical evaluation.

### Identification of BPR1K871 as a potent dual FLT3/AURKA inhibitor

Initially, we focused on investigating the effect of substitution in the 6- and 7-positions of the quinazoline ring of 4 for AURKA and FLT3 inhibition (SAR-I; Table 1). Removal of both the methoxy groups from 6- and 7-positions resulted in decreased FLT3 (over 10-fold) and AURKA (3-fold) inhibition for 5, as compared to 4. Based on the information that substitution is essential at 6-/7- positions of the quinazoline ring, 6 was synthesized bearing substitutions that are present in the marketed drug erlotinib [16]. Compound 6 with an alkoxy side chain (–OCH_2_CH_2_OCH_3_) at both 6- and 7-positions displayed similar levels of FLT3/AURKA inhibitory activities to that of 4. However, when the alkoxy side chain was present only at the 6-position (7), the inhibitory activity decreased by 10-fold for FLT3; while 8 with the alkoxy side chain at the 7-position retained the FLT3 inhibitor activity, similar to that of 4. Both 7 and 8 showed only a 2-fold decrease of AURKA inhibition levels, as compared to 4.

**Table 1:** SAR investigation at 6- and 7-position of quinazoline ring (SAR-I)

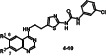										
No	R1	R2	Molecular weighta	LogD7.4a	AURKAIC50 (nM)b	AURKB Inhibition @ 500 nMc	FLT3 IC50 (nM)b	MOLM-13 EC50 (nM)b	MV4-11 EC50 (nM)b	Detroit-551 EC50 (μM)b
**4**	OMe	OMe	484.96	4.41	4.9	100%	127	20	43	9.1
**5**	H	H	424.91	4.73	15	92%	1408	69	65	6.6
**6**	O(CH_2_)_2_OCH_3_	O(CH_2_)_2_OCH_3_	573.07	4.32	6	70.4%	98	90	102	3.5
**7**	O(CH_2_)_2_OCH_3_	H	498.99	4.52	10	42%	1286	412	342	8.0
**8**	H	O(CH_2_)_2_OCH_3_	498.99	4.52	11	85.9%	103	8	9.5	2.9
**9**	H	O(CH_2_)_2_N(CH_3_)_2_	512.03	3.26	17	90.1%	58	40	57	3.4
**10 (BPR1K871)**	H	O(CH_2_)_3_N(CH_3_)_2_	526.06	2.80	22	13^b^	19	5	4	4

aMolecular weight and LogD7.4 values were calculated using MarvinSketch 15.3.16.0 (refer to Supplementary Table S1, supporting information for other Lipinski's rule of 5 parameters). bIC50 and EC50 values represent the mean of at least two independent experiments and are within ±15%. Each experiment is performed with eight concentrations, and each assay point was determined in duplicate or triplicate. c% inhibition at 500 nM; duplicate determination.

Furthermore, cellular anti-proliferative activities of the quinazoline analogues 4–8 against FLT3-ITD–expressing MOLM-13 and MV4-11 AML cell lines were evaluated by MTS assays. In particular, 8 displayed single-digit nanomolar potency, which suggests that the alkoxy ether group (–OCH_2_CH_2_OCH_3_) at the 7-position is essential and critical for dual FLT3/AURKA enzymatic inhibition, as well as cellular anti-proliferative activity.

Even though 8 displayed improved activity in both enzymatic and cellular assays, it still possessed high lipophilicity (LogD_7.4_: 4.52) and low aqueous solubility (0.107 μg/mL). With the aim of decreasing the lipophilicity, a polar amino solubilizing group was introduced at the 7-position of the quinazoline ring through a two or three carbon alkoxy linker to replace the methoxy group. Introduction of a suitable polar amino solubilizing group has been shown to improve both the physicochemical properties and inhibitor activity of several series of kinase inhibitors [17], including AURK inhibitors previously reported by us [18].

Initially, a *N*,*N*-dimethyl amino solubilizing group was introduced through a two carbon atom linker to give 9, which showed similar AURKA activity and improved FLT3 activity. When the linker length was increased from two to three carbon atoms, the corresponding analogue 10 (BPR1K871) exhibited approximately a 5-fold enhancement in FLT3 inhibition and a 2-fold decrease in AURKA inhibition, as compared to 8. Overall, it appears that the presence of an amino group (9 and BPR1K871) is better suited for FLT3 inhibitory activity than an ether (8) group when placed through a two or three carbon atoms linker at the 7-position of the quinazoline ring. Next, the anti-proliferative activities of 9 and BPR1K871 were determined in MOLM-13 and MV4-11 AML cell lines. Compound BPR1K871 with similar levels of AURKA and 3-fold enhanced FLT3 inhibitions showed approximately 7 to 14-fold enhanced anti-proliferative activities in MOLM-13 and MV4-11 AML cell lines, as compared to 9. Compound BPR1K871 with ionizable amino solubilizing group was identified as a potent dual FLT3/AURKA inhibitor possessing nanomolar efficacy in both AML cell lines.

Next, SAR exploration was carried out by retaining the *N*,*N*-dimethyl containing solubilizing group at the 7-position of the quiniazoline ring, and exploring substitution at the phenyl ring of the urea side chain attached to the 4-position of the quinazoline core. To investigate the effect of substitutions on the terminal phenyl ring of urea side chain, compounds 11–14 were synthesized and tested. (SAR-II; Table 2) Either removal of Cl group (11) or replacement with electron donating -OMe group (12) resulted in no improvement in kinase inhibitor activity or anti-proliferative activity, as compared to 10. Moreover, shifting the chloro group from the meta to ortho or para position or replacing with another electron withdrawing group (F) did not result in improvement in activity (data not shown). However, when an additional –OMe (13) or –Me group (14) was introduced at the ortho position in the phenyl ring of 10, the resulting compounds 13 and 14 showed selective FLT3 inhibition, as compared to 39. Moreover, evaluation of both the un-substituted (11), mono-substituted (12) and di-substituted (13 and 14) compounds for anti-proliferative activities in MOLM-13 and MV4-11 cells showed that the majority of the compounds exhibited single digit nanomolar inhibition.

**Table 2:** SAR investigation in the phenyl ring of urea side chain (SAR-II)

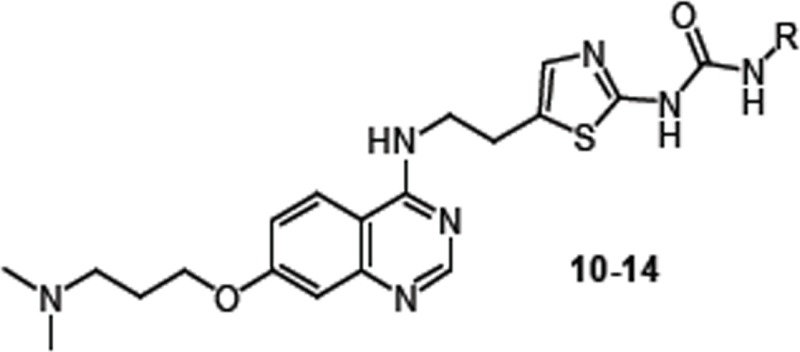									
No	R	Molecular weighta	LogD7.4a	AURKA IC50 (nM)b	AURKB Inhibition @ 500 nMc	FLT3 IC50 (nM)b	MOLM-13 EC50 (nM)b	MV4-11 EC50 (nM)b	Detroit-551 EC50 (μM)b
10 (BPR1K871)	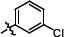	526.06	2.80	22	13b	19	5	4	4
11	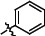	491.61	2.19	6	75.8%	94	8	21.4	10.9
12	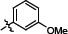	521.64	2.03	19	90%	98	12.4	10.9	9.8
13	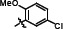	556.08	2.64	464	48.6%	16	6.7	2.1	5.3
14	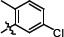	540.08	3.31	131	63%	20.5	12.7	3.3	3.8

^a^Molecular weight and LogD_7.4_ values were calculated using MarvinSketch 15.3.16.0 (refer to Supplementary Table S1, supporting information for other Lipinski's rule of 5 parameters). ^b^IC_50_ and EC_50_ values represent the mean of at least two independent experiments and are within ±15%. Each experiment is performed with eight concentrations, and each assay point was determined in duplicate or triplicate. ^c^% inhibition at 500 nM; duplicate determination. ^d^Inhibition < 50% at 1 μM.

Through detailed SAR, we identified quinazoline inhibitors with three distinct selectivity profiles - dual FLT3/AURKA (BPR1K871) selective, AURKA selective (5, 7) and FLT3 selective (13) analogs. Most of the analogs also showed inhibition of AURKB with >50% inhibition at 500 nM concentration. Importantly, many of these analogs demonstrated significant growth inhibition potential in against both MOLM-13 and MV4-11 cells; however, they were much less effective against the growth of Detroit 551 (human normal skin fibroblast cells) with EC_50_ values in micromolar level. This indicates that the quinazoline inhibitors have good therapeutic indices and selectively inhibit the growth of tumor cells, with respect to that of somatic cells.

### Molecular modeling studies of BPR1K871 with AURKA and FLT3 kinases

**Figure 2:**
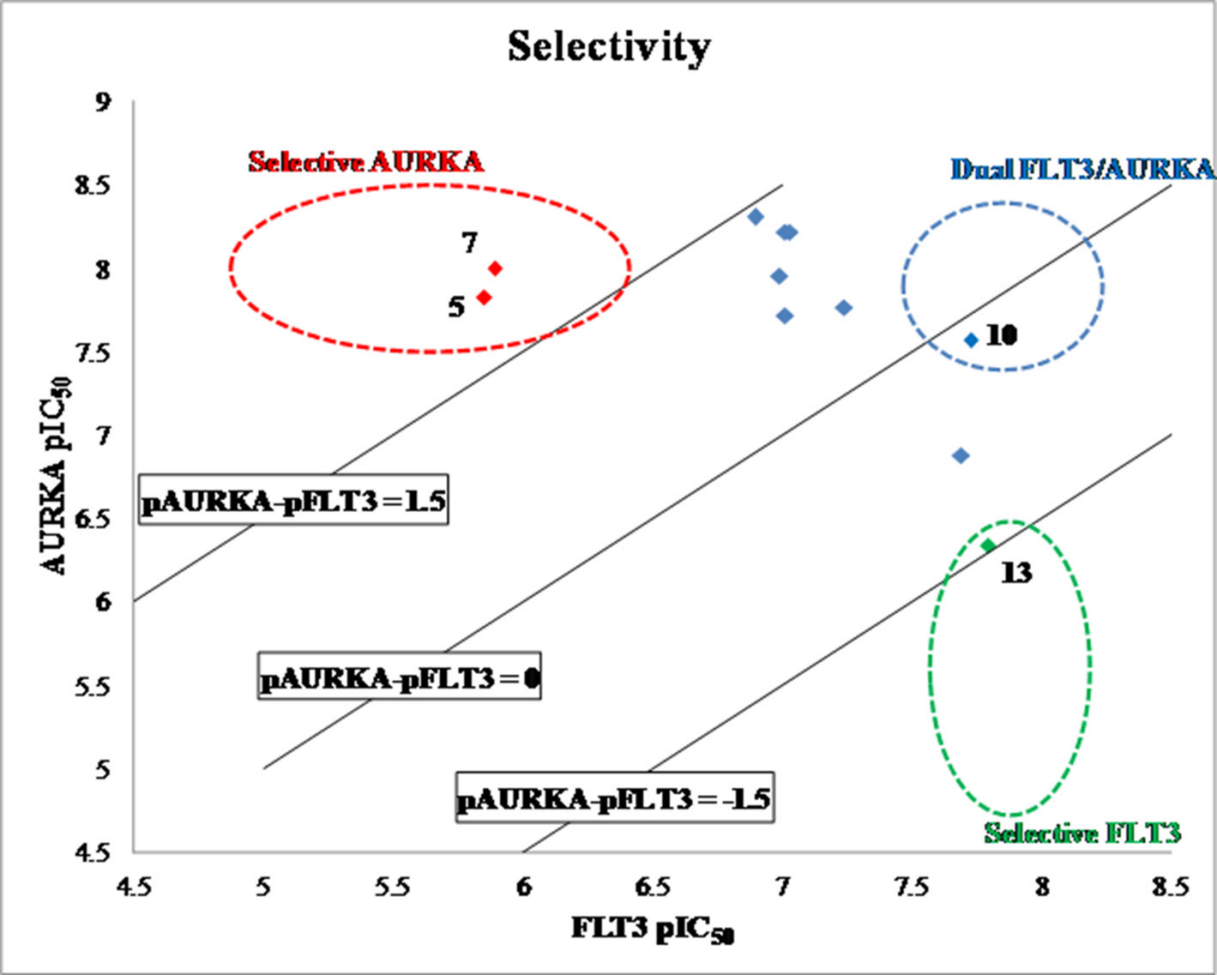
Scatter plot of FLT3 vs AURKA enzyme inhibition (data expressed as pIC50) for synthesized compounds Blue data points show dual FLT3/AURKA inhibitors, green data point shows selective FLT3 inhibitor, and red data points show selective AURKA inhibitors.

To understand the selectivity of the quinazoline inhibitors between the AURKA and FLT3 kinases, we analyzed the enzyme activity trends using a scatter plot of pIC_50_ and carried out docking studies of selected inhibitors in the active site of the enzymes. As shown in Figure 2, the compounds clustered into three groups - dual FLT3/AURKA inhibitors (blue), selective FLT3 inhibitors (green), and selective AURKA inhibitors (red). Most of the synthesized compounds were classified as dual FLT3/AURKA inhibitors. Among them, BPR1K871 (10) with an ionizable amino solubilizing group linked by a three carbon atoms linker to the 7-position of quinazoline ring is a promising dual inhibitor with excellent IC_50_ values for FLT3/AURKA kinase inhibition. While compound 13 is the most selective FLT3 inhibitor (∼30-fold selectivity over AURKA), compounds 5 and 7 are the most selective AURKA inhibitors (∼100-fold selectivity over FLT3) among the compounds synthesized in this study.

**Figure 3:**
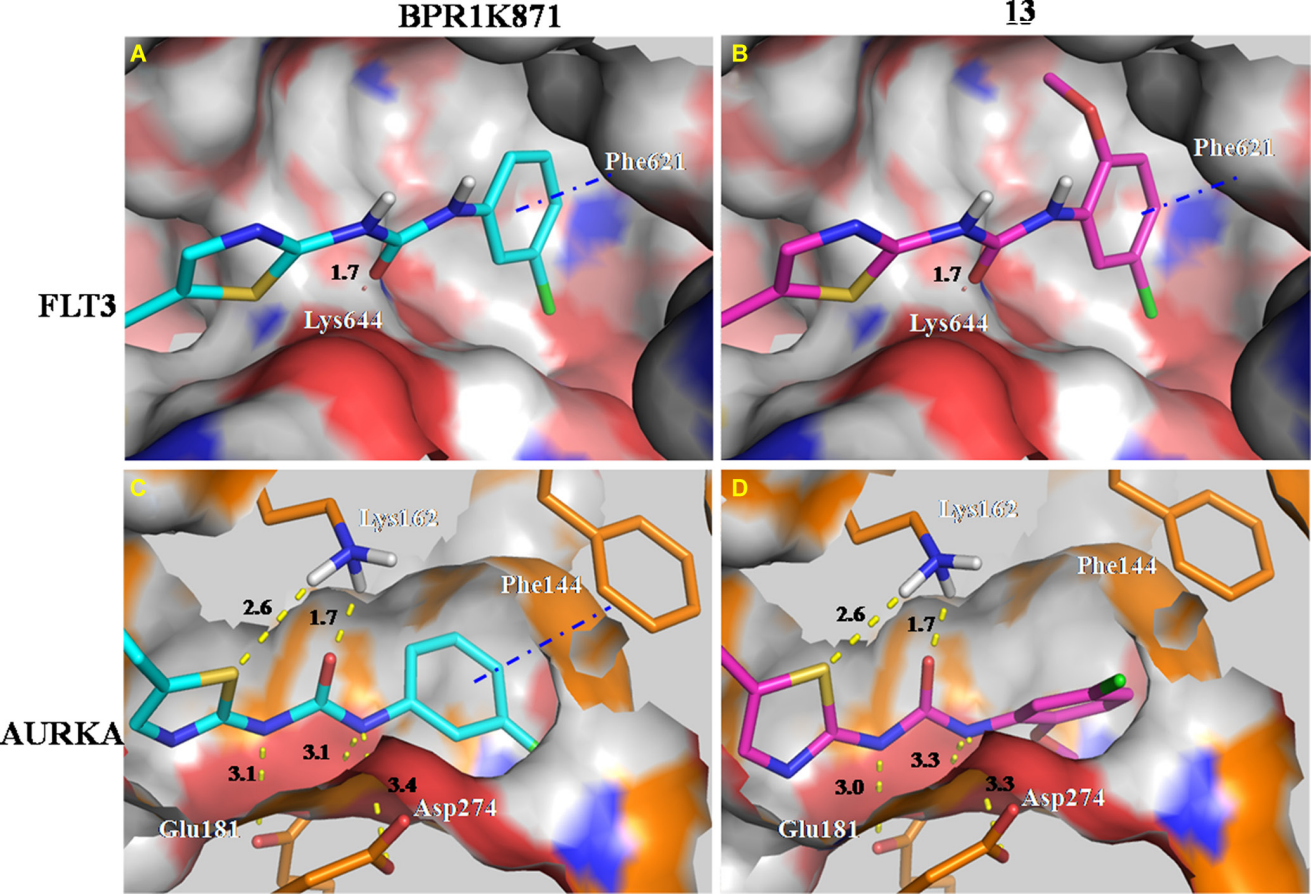
Docking study of compounds BPR1K871 and 13 in the FLT3 homology model and AURKA co-crystal (PDB ID: 4JBO) Presence of additional *ortho*-methoxy group in 13 results in loss of a critical π-π interaction with Phe144 of AURKA, making it as a FLT3 selective inhibitor.

Compound 13, a selective FLT3 inhibitor, was obtained by introducing an *ortho*-methoxy group in the terminal phenyl ring of the thiazole urea side chain of BPR1K871 (dual FLT3/AURKA inhibitor). In order to get structural insight into the shift from dual inhibition to selective FLT3 inhibition, molecular docking studies of BPR1K871 and 13 in the FLT3 homology model [13, 19] and AURKA kinase co-crystal (PDB ID: 4JBO) [20] were conducted (Figure 3) to reveal that the terminal phenyl ring of the thiazole urea side chain of BPR1K871 and 13 took a similar orientation and formed crucial π-π stacking interactions with the phenyl ring of Phe621 in the FLT3 back pocket (Figure 3A, 3B). However, in AURKA, due to a smaller back pocket region, the terminal phenyl ring of 13 was flipped by > 60° (Figure 3D) relative to the terminal phenyl ring of BPR1K871 (Figure 3C), resulting in loss of critical π-π interactions for 13 with Phe144. In contrast to 13, compound BPR1K871 was able to maintain the critical π-π interaction with Phe144 in the AURKA back pocket (due to less steric bulk at the terminal phenyl), resulting in dual FLT3/AURKA activities. Thus, presence or absence of a steric *ortho*-group at the terminal phenyl ring of the thiazole urea side chain could be helpful in fine tuning the AURKA inhibitory activity in this series of compounds. This is further emphasized by the inhibitor 14 which has an *ortho*-methyl group at the terminal phenyl ring and shows ∼8-fold selectivity for FLT3, as compared to AURKA inhibition. In addition to the observed π-π interaction, BPR1K871 formed two hydrogen bonds with FLT3 (Lys644 and Cys694) and six hydrogen bonds with AURKA (Arg137, Lys162, Ala213 and Asp274). Also hydrophobic contacts with FLT3 (Leu616, Phe621, Ala642, Leu658, Gly661, Glu692, Tyr693, Cys695, Try696, Gly697, Leu818, Cys828, Asp829 and Gly831) and AURKA (Leu139, Val147, Leu149, Ala160, Leu164, Val174, Leu178, Leu194, Leu210, Tyr212, Pro214, Leu215, Thr217, Glu260, Leu263, Ala273, Gly276 and Trp277) were observed.

Next, AURKA selective inhibitors 5 and 7 without polar ionizable amino substituents at the 7-position of quinazoline ring displayed ∼100-fold selectivity for AURKA over FLT3, compared to the dual selective inhibitor BPR1K871. To investigate the impact of this side chain, BPR1K871 and 5 were docked to an FLT3 homology model and the binding interactions were analyzed using LIGPLOT software [21] (see Supplementary Figure S1, Supporting Information). Both BPR1K871 and 5 showed a hydrogen bond between the quinazoline ring N1 and Cys694 in the kinase hinge region as well as H-bond between the urea function and Lys644. In addition to the H-bonds, the 7-position side chain of BPR1K871 formed a number of hydrophobic contacts with the residues Cys695, Tyr696, and Gly697 resulting in significantly enhanced FLT3 inhibition. In order to further verify our observation, binding energy calculations of BPR1K871 and 5 were also performed. Compound 5 has a less favorable FLT3 binding energy compared with BPR1K871 (-45.75 kcal mol^-1^ vs. –80.27 kcal mol^-1^), which is also consistent with the IC_50_ findings. Overall, removal of the 7-alkoxyl group causes significant loss of FLT3 activity, to make quinazoline analogs 5 and 7 as AURKA selective inhibitors.

### Investigation of BPR1K871 as a potent anti-AML agent with *in vivo* efficacy in tumor xenograft models

Through detailed SAR and computer modeling studies, we identified quinazoline BPR1K871 as a potent dual FLT3/AURKA inhibitor with anti-proliferative activities in MOLM-13 and MV4-11 AML cell lines, with single digit nanomolar IC_50_. Both the cell lines are FLT3-ITD mutation positive AML cell lines. In addition, we tested the anti-proliferative ability of BPR1K871 in three other leukemia cell lines - U937 an AML-cell line which is FLT3 negative, RSV-11 cell line which is an ALL cell line with wt-FLT3 and in CML cell line K562 expressing Bcr-Abl fusion protein (Table 3). For comparison purpose known inhibitors VX680 and barasertib (AURK inhibitors), linifanib, sorafenib, and PKC412 (multi-kinase inhibitors) were also tested in these cell lines side-by-side for anti-proliferative activity. In the FLT3 expressing cell lines (MOLM-13, MV4-11 and RS4-11), quinazoline BPR1K871 showed low nanomolar IC_50_ which was better than the standard inhibitors tested. However, the anti-proliferative activity was in low micromolar range in leukemia cell lines which are FLT3 negative (U937 and K562), suggesting that BPR1K871 could act through FLT3 target inside the cell.

**Table 3:** Anti-proliferative activity of BPR1K871 on a panel of in-house leukemia cell lines

Cell line	Cell type	EC_50_ (nM)^a^					
		BPR1K871	Linifanib	Sorafenib	PKC412	Barasertib	VX-680
MOLM-13	AML-FLT3-ITD(heterozygous)	5	38	82	55	42	69
MV4-11	AML-FLT3-ITD(homozygous)	4	82	43	38	17	71
RS4-11	ALL-wt-FLT3(homozygous)	11	9200	9300	400	11	nd
U937	AML-FLT3-negative	8050	>18000^b^	3350	1400	>10000^c^	nd
K562	CML-Bcr-Abl FLT3-negative	2300	>20000^d^	7300	>20000^d^	>10,000^c^	nd

^a^EC_50_ values represent the mean of at least two independent experiments performed with eight concentrations, and each assay point was determined in duplicate or triplicate. Refer to Supplementary Table S2, supporting information for SEM data. ^b^Inhibition < 50% at 18 μM. ^c^Inhibition < 50% at 10 μM. ^d^Inhibition < 50% at 20 μM. nd – not determined.

**Figure 4:**
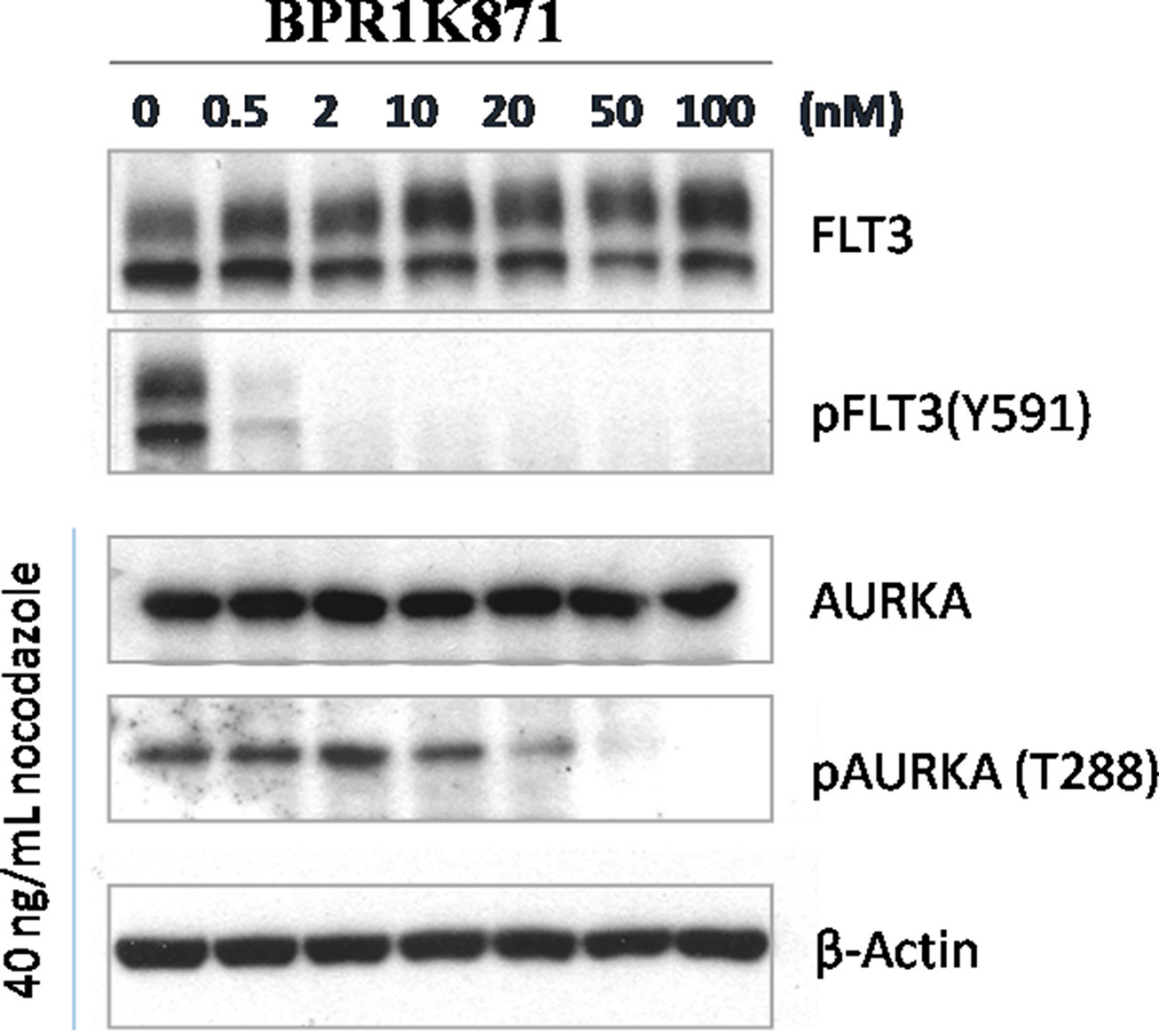
Western blot analysis for cellular target modulation by BPR1K871 Phospho-FLT3 (Tyr591) and phospho-AURKA (Thr288) formation in MV4-11 cells were completely inhibited at concentrations of 2 and 100 nM, respectively.

In order to understand the mode of action of BPR1K871, mechanistic studies in MV4-11 cell line using western blot analysis was carried out. MV4-11 cells were incubated with BPR1K871 in a dose-dependent manner for 2.0 h. Cell lysates were prepared and analyzed by immunoblotting for both FLT3 phosphorylation (pFLT3 at residue Y591) and AURKA phosphorylation (pAURKA at residue T288). It was observed that BPR1K871 at 2 and 100 nM completely inhibited the formation of pFLT3 and pAURKA, respectively, which suggest that the anti-proliferative activity of BPR1K871 is due to FLT3/AURK target modulation inside the cells (Figure 4).

**Figure 5:**
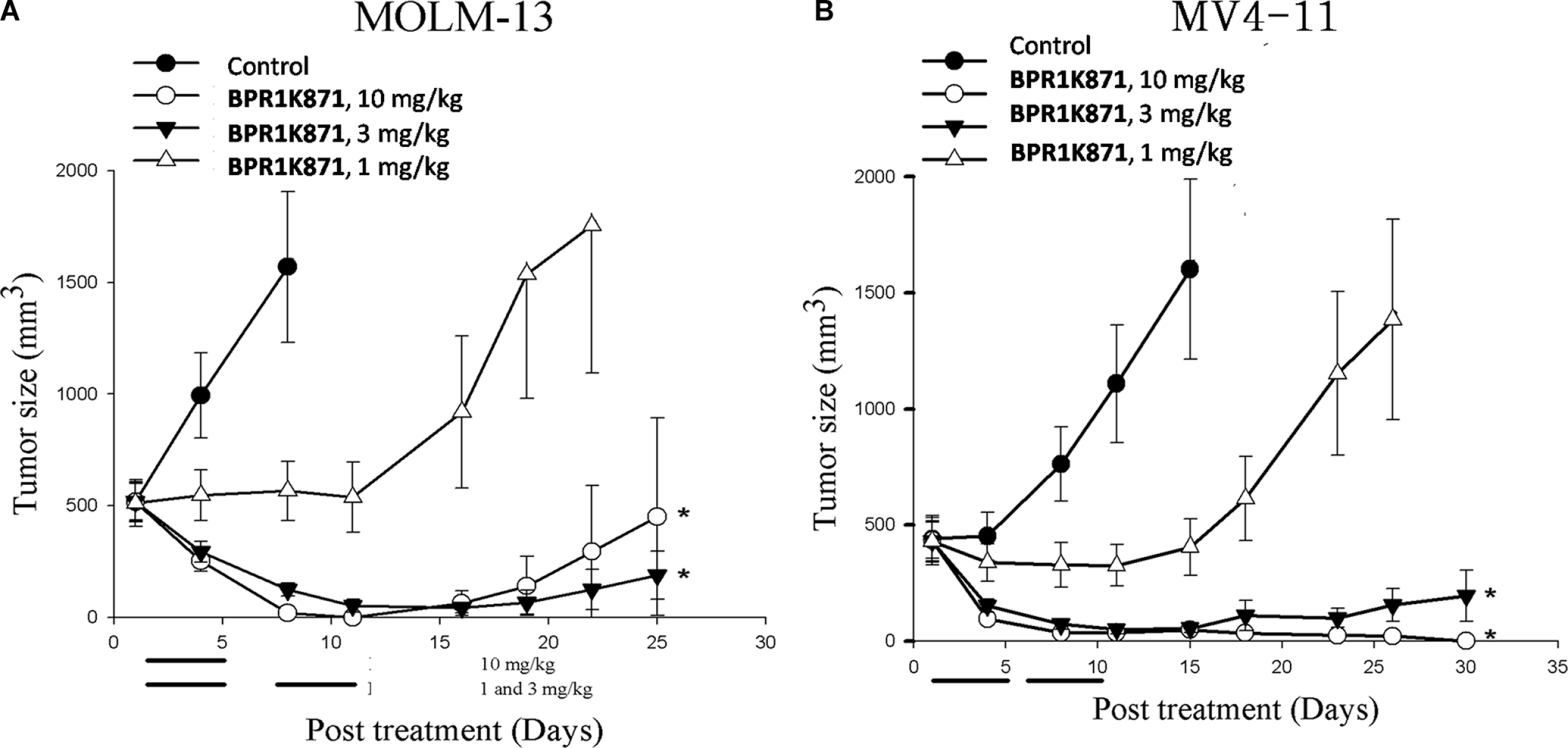
In vivo antitumor effect of BPR1K871 in human acute myelogenous leukemia xenograft nude mice model (**A**) The growth of MOLM-13 tumor xenograft is inhibited by BPR1K871 (1, 3, or 10 mg/kg, iv) with *p* < 0.05. Drug treatment on days 1–5 for all groups and 8–12 for groups of 1 and 3 mg/kg. (**B**) The growth of MV4-11 tumor xenograft is inhibited by BPR1K871 (1, 3, or 10 mg/kg, iv) with *p* < 0.05. Drug treatment on days 1–5 and 8–12.

Based on the potent *in vitro* anti-proliferative activity of BPR1K871, it was planned to investigate the *in vivo* efficacy. For choosing the appropriate route and schedule for drug administration, *in vivo* pharmacokinetic (PK) properties of BPR1K871 were determined in the rat by iv administration; a long t_1/2_ and moderate PK profile, albeit without oral bioavailability, was observed (Supplementary Table S3, Supporting Information). Subsequently, based on the PK study, we evaluated the acute toxicity of BPR1K871 in ICR mice (*n* = 3) by iv administration. It was found that BPR1K871 can be well tolerated at 50 mg/kg administered once a day for five days. Next, the antitumor activity of BPR1K871 was evaluated through IV route in MOLM-13 and MV4-11 xenograft models (Figure 5). For this, nude mice were inoculated subcutaneously with MOLM-13 and MV4-11 cells in the left flank. When the tumor size reached approximately 500 mm^3^, BPR1K871 was intravenously administered to suppress the growth of the subcutaneously xenografted tumors. The hydrochloride salt of BPR1K871 was dissolved in a formulation of DMSO/cremophor EL/saline (10/20/70) and administered at three different doses. For MOLM-13 model, BPR1K871 was injected intravenously at 10 mg/kg daily for 5 days (on days 1–5); also BPR1K871 was intravenously administrated at 3 and 1 mg/kg once a day for two continuous weeks (on days 1–5 and 8–12). For MV4-11 model, BPR1K871 was intravenously administrated at 10, 3 and 1 mg/kg once a day for two continuous weeks (on days 1–5 and 8–12). The test compound BPR1K871 at 3 and 10 mg/kg significantly reduced the volume of the subcutaneously xenografted MOLM-13 and MV4-11 tumor as compared to vehicle-treated controls in nude mice.

### Investigation of BPR1K871 as a potent multi-kinase inhibitor with *in vivo* efficacy in solid tumor xenograft models

**Figure 6:**
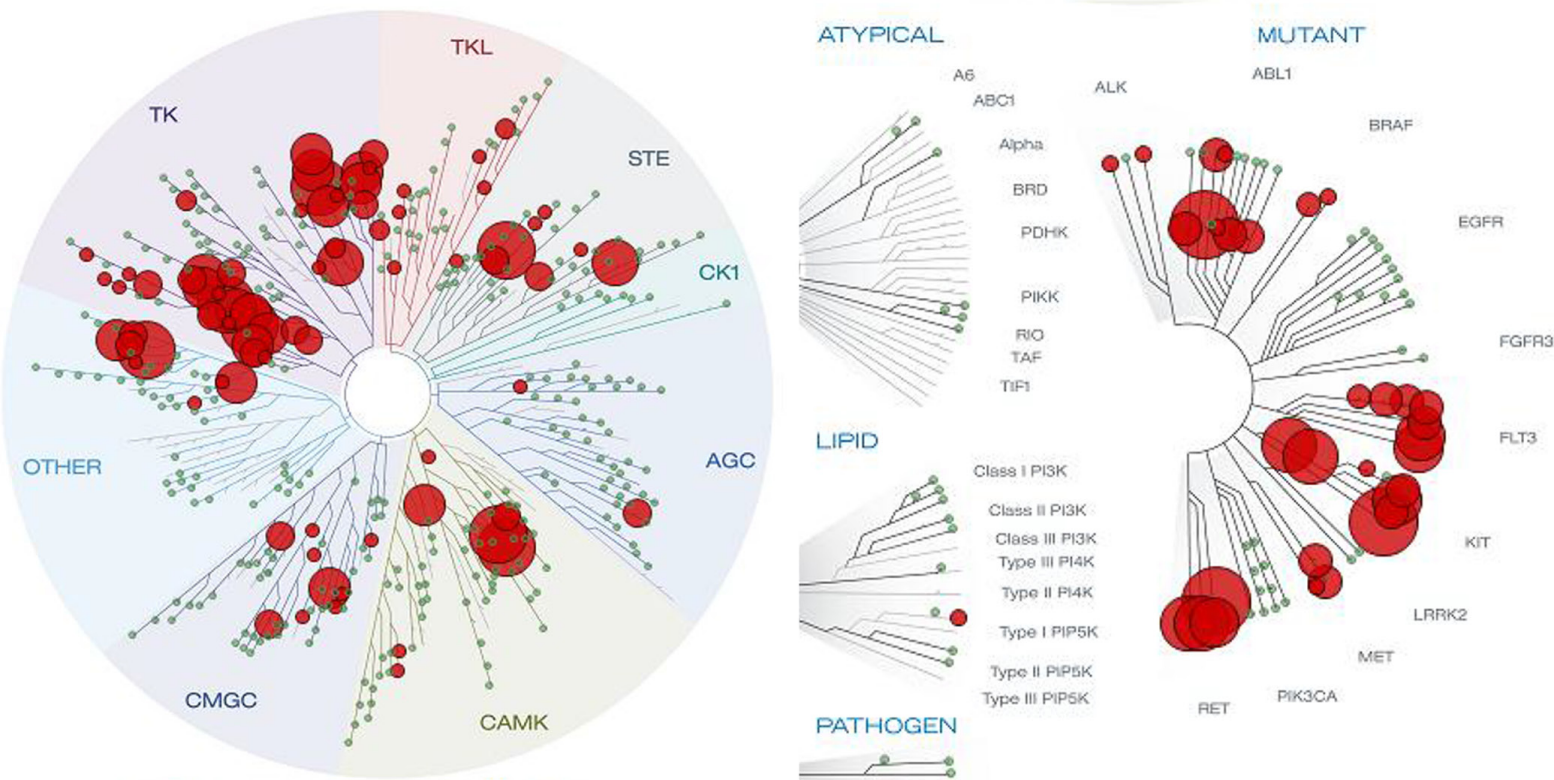
Kinome tree view depicting the kinase selectivity of BPR1K871 as determined by KINOMEScan at 1 μM concentration BPR1K871 is a multi-kinase inhibitor targeting several cancer-associated kinase and their mutant forms.

Once BPR1K871 was identified as a potent anti-leukemia agent with *in vivo* activity, we were interested to develop it further. For this purpose, BPR1K871 was initially examined using the KINOMEScan technology against a panel of 456 kinases (containing 395 non-mutant kinases) at a concentration of 1000 nM [22]. AURKA, AURKB, AURKC, and FLT3 were potently inhibited by BPR1K871 (0, 0.2, 3.3, and 0.2% control, respectively, at 1000 nM). In addition, BPR1K871 also exhibited high affinity against clinically relevant FLT3 mutants (< 5.2% control for FLT3^ITD^, FLT3^D835H^, FLT3^D835Y^, FLT3^K663Q^, FLT3^N841I^, and FLT3^R834Q^ at 1000 nM). Furthermore, BPR1K871 has a broad spectrum of activity for other tumor associated kinases including ABL1, AXL, BRAF, CHEK2, CSF1R, DDR, FLT1, KIT, PDGFR, PLK4, RET, TRKA, VEGFR2, etc. (Supplementary Table S4, Supporting Information); the S(35) selectivity score was determined as 0.197, which is calculated using <35% of control as a potency threshold at a concentration of 1 μM. Moreover, clinically relevant ABL1 mutants (< 4.4% control for ABL1^T315I^, ABL1^Q252H^, ABL1^H396P^), KIT mutants (< 4.0% control for KIT^L576P^, KIT^V559D^, KIT^V559D,T670I^, KIT^A829P^, KIT^V559D,V654A^) and RET mutants (< 0.5% control for RET^V804M^, RET^M918T^, RET^V804L^) were also inhibited potently. The KINOMEScan result clearly demonstrates that BPR1K871, identified through scaffold-hopping from a furanopyrimidine core to a quinazoline core followed by SAR exploration, not only possesses excellent dual FLT3/AURK activities but is also a multi-kinase inhibitor (Figure 6).

Based on the activity of BPR1K871 against several cancer-associated kinases, we next examined the cyototoxicity spectrum of compound BPR1K871 against a panel of human solid tumor cell lines (Table 4). Growth inhibitory activities of reported multi-kinase inhibitors (linifanib, sorafenib, and PKC412) [23], and AURK inhibitors (barasertib and VX-680) [10] are also provided for reference. The results reveal that BPR1K871 inhibited proliferation against tested cancer cells with EC_50_ values ranging from 34 nM to 7 μM. Among these, treatment with BPR1K871 induced significant cell death against colon (COLO205), and pancreatic (Mia-PaCa2) cell lines (EC_50_ values < 100 nM), with better potency compared to the reference inhibitors.

**Table 4:** Anti-proliferative activity of BPR1K871 on a panel of in-house non-leukemia cancer cell lines

Cell Line	Cell type	EC_50_ (nM)^a^					
		BPR1K871	Linifanib	Sorafenib	PKC412	Barasertib	VX-680
HCC827	NSCLC-EGFR^L858R^	257	6182	6273	687	127	nd
H1975	NSCLC-EGFR^LR/TM^	495	8853	7010	517	390	nd
H2228	NSCLC-ALK	212	8785	7973	609	340	nd
CL-97	Human Lung Cancer	2891	>20000^d^	7054	916	454	nd
HCT-116	Human Colon Cancer	141	8070	8800	482	67	97
Colo 205	Human Colon Cancer	34	nd	nd	nd	nd	86
Mia-PaCa2	Human Pancreatic Cancer	94	16369	7792	995	nd	nd
MESSA	Uterine Sarcoma	216	nd	nd	nd	nd	nd
MESSA/DX	Doxorubicin Drug Resisted MESSA	6900	nd	nd	nd	nd	nd
Hep 3B	Human Hepatoma	2471	nd	nd	nd	nd	nd
CL 1-5	Human Lung Adenocarcinoma	1387	nd	nd	nd	nd	nd
MKN-45	Human Gastric Cancer	355	nd	nd	nd	nd	nd

^a^EC_50_ values represent the mean of at least two independent experiments performed with eight concentrations, and each assay point was determined in duplicate or triplicate. Refer to Supplementary Table S5, supporting information for SEM data. ^b^Inhibition < 50% at 18 μM. ^c^Inhibition < 50% at 10 μM. ^d^Inhibition < 50% at 20 μM. nd – not determined.

**Figure 7:**
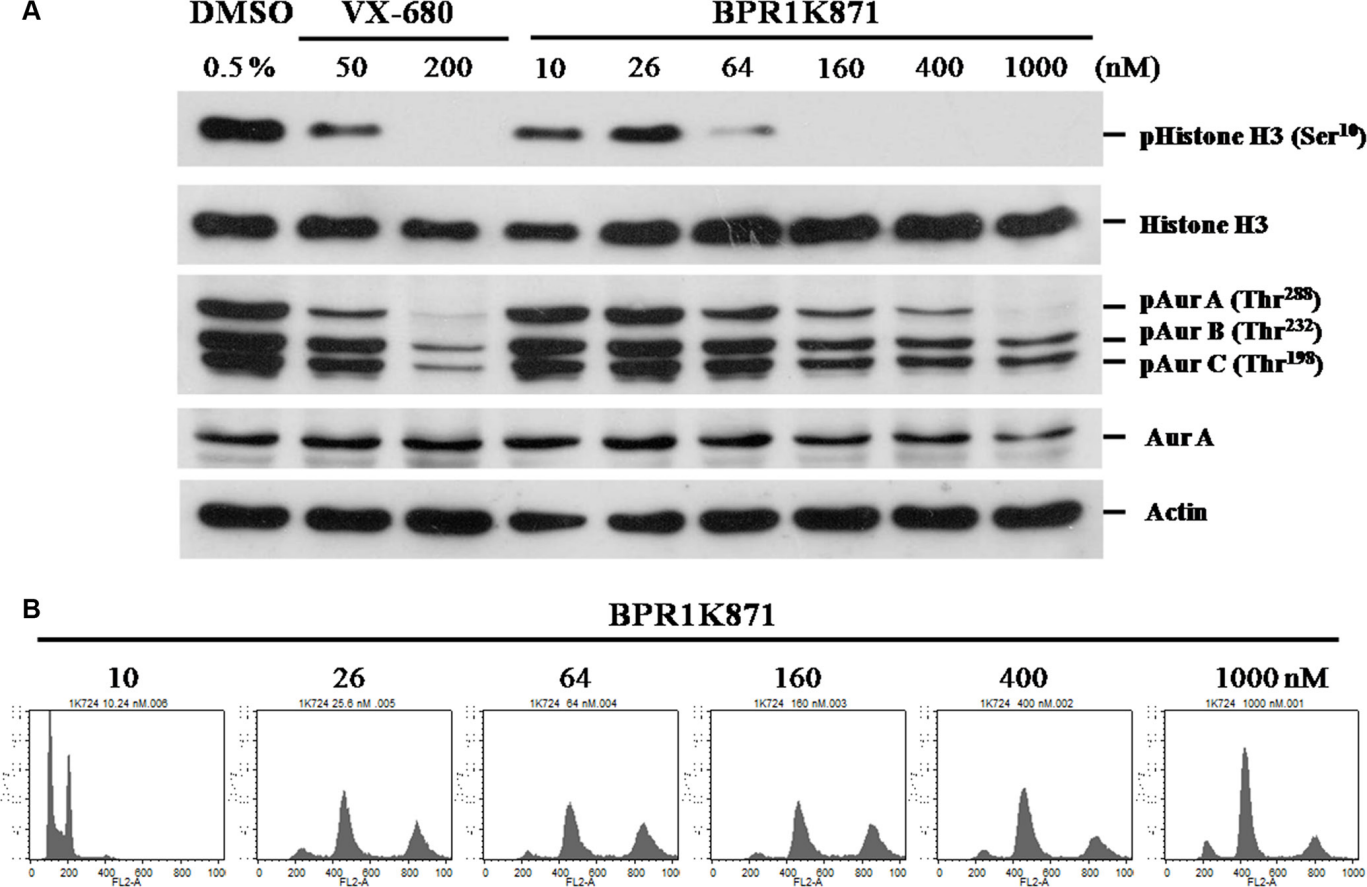
Functional study of BPR1K871 on mitotic progression in HCT-116 cell line (**A**) Western blot analysis of HCT-116 cell lines treated with various concentrations of BPR1K871 showing decreased levels of phospho-histone H3, phospho-AURKA (Thr288), phospho-AURKB (Thr232) and phospho-AURKC (Thr198). VX-680 is used as positive control. (**B**) DNA content analysis using flow cytometry of HCT-116 cells treated with BPR1K871. Multinuclealed cells were identified with 4N and 8N duplicated DNA content at concentration of 26 nM onwards.

Next, western blot analysis to determine the inhibition of phosphorylation of Aurora substrates in HCT-116 cells treated with BPR1K871 for 2.0 h was carried out. BPR1K871 inhibited phospho AURKA (Thr288), AURKB (Thr232), AURKC (Thr198), and phospho histone H3 (Ser10) formation in HCT-116 cells in a dose dependent manner (Figure 7A). In addition, accumulated multinucleated cells with 4N or 8N DNA content were observed in a flow cytometry analysis of HCT-116 cells treated with compound BPR1K871 at a concentration of 26 nM for 48 h, an indicator of mitotic checkpoint override by the inhibition of AURKB (Figure 7B). These experiments suggest potent *in vitro* efficacy of BPR1K871 to pan-AURK inhibition.

The KINOMEScan results and *in vitro* activities in different cell lines encouraged us to assess the *in vivo* anti-cancer activity of BPR1K871 in solid tumor models. For this, COLO205 and Mia-PaCa2 xenograft models were established through inoculating nude mice subcutaneously with corresponding cancer cells. When the tumor size reached approximately 200 mm^3^, the hydrochloride salt of BPR1K871 dissolved in the formulation was intravenously administered to suppress the growth of the subcutaneously xenografted tumors. As shown in Table 5, tumor regression for COLO205 and Mia-PaCa2 were observed at the tested 20 mg/kg dose level without body weight loss of more than 15%; no mice died during the study. Overall, the *in vivo* results suggest that the multi-kinase inhibitor BPR1K871 has promising anti-cancer efficacy without observed toxicity, and would be a potential anti-cancer candidate against not only AML but also solid tumors.

**Table 5:** Efficacy of BPR1K871 in tumor xenograft models in vivo^a^

Xenograft model	*n* =	Dose (mg/kg)	TGI ± SD^b^
COLO205	8	20	93 ± 9%
Mia-PaCa2	9	20	91 ± 10%

^a^Subcutaneous COLO205 or Mia-PaCa2 tumors in nude mice (size of ∼200 mm^3^) were treated with intravenous administration of BPR1K871 once a day for two continuous weeks (on days 1–5 and 8–12). ^b^Tumor growth inhibition (TGI) was calculated using the following formula: TGI = [1 – T / C] × 100, where T indicates the mean tumor volume (mm^3^) of the test groups and C indicates the mean tumor volume (mm^3^) of the vehicle-treated group, COLO205 (day 15); Mia-PaCa2 (day 29).

### Synthesis of BPR1K871

**Figure 8:**
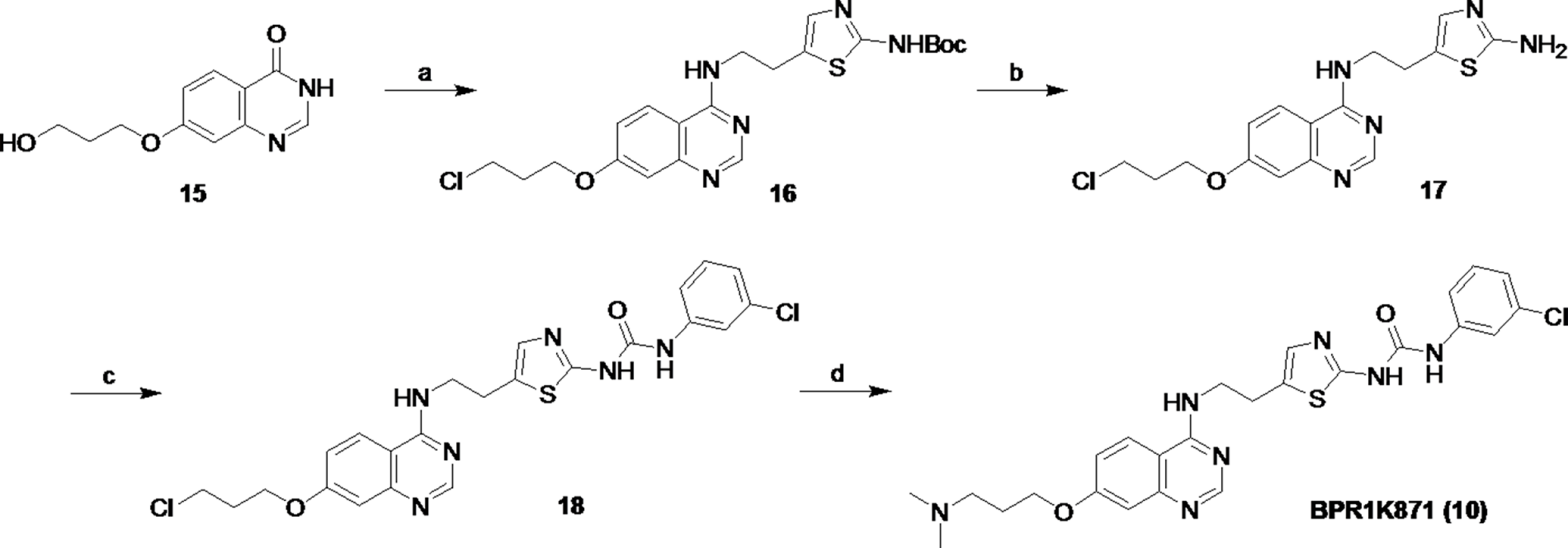
Synthetic route for BPR1K871 (a) (i) SOCl_2_, reflux, 15 h, (ii) tert-butyl (5-(2-aminoethyl)thiazol-2-yl)carbamate, Et_3_N, EtOH, reflux, 15 h, 58% for two steps; (b) CF_3_CO_2_H, CH_2_Cl_2_, rt, 12 h, 98%; (c) 3-Cl-PhNCO, MeOH, CH_2_Cl_2_, rt, 16 h, 63%; (d) dimethylamine, KI, DMF, 100°C, 3.0 h, 36%.

BPR1K871 was synthesized as shown in Figure 8. For this purpose, quinazolinone 15, prepared according to previous methods [24–27], was used as starting material. The intermediate 16 was synthesized from the quinazolinone 15 by chlorination using thionyl chloride, followed by nucleophilic aromatic substitution with *tert*-butyl (5-(2-aminoethyl)thiazol-2-yl)carbamate [28]. After Boc deprotection under acidic conditions, intermediate aniline 17 was subjected to urea formation with 3-chlorophenyl isocyanate to afford the intermediate 18. Consequently, nucleophilic substitution of the chloro compound 18 by dimethylamine in DMF gave the desired BPR1K871 (10).

## DISCUSSION

Since the launch of the first kinase targeted cancer therapeutics – imatinib for CML treatment, several kinase targeted small molecule inhibitors and monoclonal antibodies are approved for the treatment of various cancers [16, 29, 30]. The rational for their development is the identification of aberrant kinase signaling specific for the cancer and targeting them with either small molecule inhibitors or using monoclonal antibodies to abrogate the aberrant signaling, thereby blocking the un-controlled proliferation. This strategy has been successful in few specific cases of cancer where a single/specific genetic alteration is the driving force for the cancer. For example, the majority of CML is caused by Bcr-Abl fusion protein expression and targeting the aberrant Abl kinase signaling by imatinib shuts down the proliferation signaling [31]. Similarly, gefinib, an approved treatment for non-small cell lung cancer target EGFR which is mutated and the cause for aberrant signaling in a set of lung cancer patients [32]. On similar lines, shutting down the signaling using monoclonal antibody trastuzumab is very successful for the treatment of breast cancer over expressing Her2 receptor [30]. However, in many of the cancers there are multiple genetic alterations driving the cancer, hence using drugs that target single genetic alterations becomes ineffective. The way out is to use a drug combination treatment regime or use agents that target multiple signaling molecules that are aberrant in the cancer [33–35].

In this regard, several drugs that target multiple kinases in the signal transduction pathway have been successfully developed in the past; these include sorafenib, sunitinib, pazopanib, vandetanib, axitinib, regorafenib, nintedanib, and the newly introduced lenvatinib [16, 29]. It is interesting to note that these multi-kinase inhibitors were found to be very effective in some previously difficult to treat malignant diseases such as renal cancer, liver cancer, metastatic colon cancer and pancreatic neuroendocrine cancer [36]. The successful launch of many multi-kinase inhibitors suggests that multi-targeted agents could play an important role in combating cancer in the future.

So far, majority of the reported kinase inhibitors target the ATP binding site and are competitive in nature, resulting in some level of cross-reactivity to a set of kinases. As discussed above this is an advantage in oncology, as multiple aberrant signaling pathways could be blocked by single agent. In fact inhibitors that were considered to be very specific for a particular kinase in the early stage of development has been later identified to target additional kinases. For example, recent large scale kinase profiling showcased the multi-kinase potential of several kinase inhibitors [37]. Several of these inhibitors were initially developed by optimizing their activity against few kinases and later found to have multi-kinase potential [34].

We have been involved in the development of kinase inhibitors as targeted anti-cancer agents and have reported series of compounds based on furanopyrimidine and quinazoline cores as potential AURK/FLT3 inhibitors [13–15, 18]. Based on the knowledge generated from previous SAR studies, here we conducted scaffold-hopping from a furanopyrimidine core (1 & 3) to a quinazoline core (4) to improve physicochemical properties such as lipophilicity (LogD_7.4_: 7.10 to 4.41), and also lower the molecular weight (567 to 485). More importantly, the quinazoline core is considered a privileged structure for the inhibition of ATP-dependent kinases, since 5 out of 30 kinase inhibitors approved by the FDA possess the quinazoline framework [16]. Based on the above facts, we undertook the synthesis and testing of quinazoline 4.

The newly designed quinazoline 4 (AURKA IC_50_ = 4.9; FLT3 IC_50_ = 127 nM) showed dual enzyme inhibition as well improved anti-proliferative activity in AML cell lines (EC_50_ ∼ 40 nM). To further improve the activity and aqueous solubility (0.452 μg/mL) of 4, two sets of compounds were synthesized by altering the functional groups at the quinazoline core (SAR-I) and at the terminal phenyl ring of the urea side chain (SAR-II). Detailed SAR exploration of the quinazoline 4 revealed that the introduction of a solubilizing amino group (*N*,*N*-dimethyl) at the 7-position of quinazoline ring is best suited for FLT3 inhibitory activity (9 and BPR1K871); compounds 5 and 7 bearing a 7-H quinazoline were identified as AURKA-selective inhibitors. The presence of a thiazole urea side chain with a terminal phenyl group was found to be critical for maintaining dual AURKA and FLT3 activities. More importantly, presence of an *ortho*-substitution in the terminal phenyl ring, independent of its electronic nature, resulted in selective FLT3 inhibition (13), which is due to the inability of the phenyl ring to form critical π-π interactions with Phe144 in AURKA. In contrast, BPR1K871 lacking the *ortho*-substitution is able to maintain the π-π interactions with Phe144 in AURKA, resulting in dual FLT3/AURKA inhibition.

**Figure 9:**
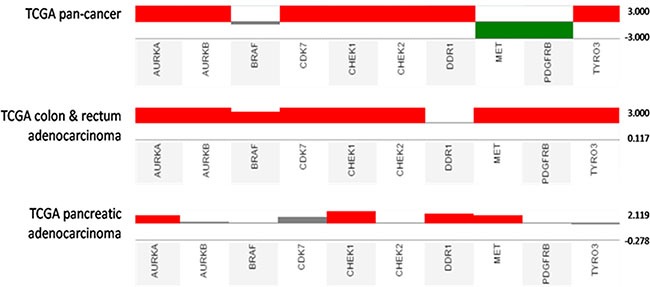
Kinase gene expression analysis in cancer tissues (TCGA pan-cancer, TCGA colon & rectum adenocarcinoma, and TCGA pancreatic adenocarcinoma) compared with normal tissues using UCSC Cancer Genomics Browser Gene expression datasets use red and green to represent over- and under-expression, respectively.

Among the compounds tested, BPR1K871 (10) bearing the polar amino solubilizing group at the 7-position of the quinazoline ring, exhibited superior dual FLT3/AURK inhibition. Furthermore, BPR1K871 was identified as a multi-kinase inhibitor with a broad inhibition potential against several cancer-associated kinases such as ABL1, AXL, BRAF, CHEK2, CSF1R, DDR, FLT1, KIT, PDGFR, PLK4, RET, TRKA, VEGFR2 besides AURK and FLT3. Also several clinically relevant mutants including FLT3 mutants (FLT3^ITD^, FLT3^D835H^, FLT3^D835Y^, FLT3^K663Q^, FLT3^N841I^, and FLT3^R834Q^), ABL1 mutants (ABL1^T315I^, ABL1^Q252H^, ABL1^H396P^, ABL1 ^F317L^), KIT mutants (KIT^L576P^, KIT^V559D^, KIT^V559D,T670I^, KIT^A829P^, KIT^V559D,V654A^, KIT^D816V^) and RET mutants (RET^V804M^, RET^M918T^, RET^V804L^) were also inhibited. Moreover, BPR1K871 inhibited AML (MOLM-13 and MV4-11), colorectal (COLO205) and pancreatic (Mia-PaCa2) cancer cell proliferation in *in vitro*, better than the standard/clinically used multi-kinase & aurora kinase inhibitors tested. Mechanistic studies in MV4-11 and HCT-116 cell lines suggest that the cancer relevant targets AURKA/FLT3 are inhibited inside the cells. It is relevant to note that the potent anti-proliferative activities in these cell lines, expressing different types of kinase, were justified by the strong inhibition of these kinases by BPR1K871. In particular, analysis of UCSC Cancer Genomics database (https://genome-cancer.ucsc.edu/) suggests that colon/rectum and pancreatic adenocarcinoma show upregulation of multiple kinases (AURKA/B, CHEK1, MET, etc) (Figure 9), which are potently inhibited by BPR1K871. Taken together, the data suggests that the multi-kinase inhibitor BPR1K871 efficiently inhibits the cancer cellular proliferation via blocking single or multiple key pathways involved in cancer.

BPR1K871 bearing the polar amino solubilizing group at the 7-position of the quinazoline ring, exhibited multi-kinase inhibitions as well as excellent cellular anti-proliferative activity in not only AML cell lines, but also in solid tumors. The two main purposes of introducing a polar amino solubilizing group are to reduce the liphophilicity (LogD), and increase the aqueous solubility. Introduction of *N*,*N*-dimethyl group led to lower LogD_7.4_ for BPR1K871 (2.80), as compared to initial lead 4 (4.41). Second, the basic amine functional group (calculated pKa = 9.21) [38] in BPR1K871 provided an effective means to increase the solubility by ionization. The aqueous solubility of BPR1K871 in pH ≤ 5.2 buffer solution was significantly improved up to 4000-fold, as compared to that of BPR1K871 in water (0.124 μg/mL). Hence, high solubility of the ionized form (ie. salt form) makes it more suitable for further evaluation in *in vivo* efficacy studies. Further, *in vivo* pharmacokinetics experiments in rats suggest that only intravenous administration of BPR1K871 is suitable for efficacy evaluation. It should be noted that several kinase inhibitors have been administrated as an iv infusion in clinical trials [39]. For example, volasertib, an intravenous polo-like kinase (PLK) inhibitor, is currently in phase III for the treatment of AML [40]. A phase II/III trial using barasertib (an intravenous AURKB inhibitor) alone and in combination with low dose cytosine arabinoside has been completed in AML [41]. Moreover, a phase II study to evaluate the efficacy and safety of intravenous PI3K inhibitor BAY80-6946 in patients with indolent or aggressive non-Hodgkin's lymphoma is currently underway [42]. Encouraged by these reports, we initiated a proof-of-concept, *in vivo* experiment to evaluate BPR1K871 through iv administration in tumor xenograft models.

The hydrochloride salt of BPR1K871 exhibited excellent *in vivo* efficacy not only in leukemia (MOLM-13 and MV4-11) but also in colorectal (COLO205) and pancreatic (Mia-PaCa2) xenograft nude mouse models after IV administration at a dose range of 3–20 mg/kg, without adverse toxicity. Considering the *in vivo* efficacy in different cancer models, particularly in pancreatic cancer, it is worthwhile to further investigate BPR1K871; pancreatic and liver cancer are the most fatal of cancers and 5-year relative survival for patients with pancreatic cancer is reported to be just 8% [43, 44]. In addition, preliminary ADME and safety evaluation of BPR1K871 using hERG inhibition assay (66% at 10 μM) [45], CYP inhibition assay (IC_50_ values greater than 4 μM for CYP 1A2, 2C9, 2C19, 2D6, 3A4, and 2E1), and microsomal stability assay (Human > 80%, Mouse > 30% remaining at 30 min), suggests that BPR1K871 is a suitable candidate for further preclinical development.

Overall, the *in vitro* and *in vivo* results suggest that BPR1K871 is a multi-kinase inhibitor which may provide therapeutic benefit over existing treatment and warrants further preclinical investigations. In many cancers multiple aberrations in signaling pathways are reported. Moreover, widespread development of drug resistance due to point mutations or gene amplification of target proteins and redundancy in the signaling pathways, makes the use of single-kinase agents ineffective and the use of a multi-kinase agents provide potential clinical advantage [34, 46]. Due to this, multi-kinase targeting is becoming a successful therapeutic strategy in the treatment of several cancers and many multi-kinase inhibitors are approved by US FDA [16, 29]. In this context, development of BPR1K871 could provide an effective anti-cancer therapeutics in the future.

## MATERIALS AND METHODS

### Docking study in AURKA and FLT3

For the docking study in AURKA, previously reported 3D Aurora A protein structure (PDB ID: 4JBO) [20] was used. While, for the docking study in FLT3, the previous reported homology modeled DFG-in FLT3 structure was used [13, 19]. The docking calculation was conducted using the DS/LigandFit program (Discovery Studio 2.1, Accelrys, Inc., San Diego, CA) with the CHARMm force field [47]. The number of docking pose was set as 50 with default parameters. The docking RMS threshold for ligand-site matching was set as 2 angstroms. The number of steepest descent steps for the rigid-body minimization during pose docking was set as 100. The decision of the best pose was according the binding information from the complex structure.

### Binding energy calculation

The binding energy calculations were performed by DS 2.5/Calculate binding energies program. A 1000-step Smart minimizer method in the Distance-Dependent Dielectric solvent model implicit solvent environment was used with the default parameter values before calculating the binding energies.

### Biological methods

#### FLT3 kinase inhibition assay

The FLT3 assay was conducted as previously described [48]. Briefly, GST-FLT3-KD^WT^ containing the FLT3 kinase catalytic domain (residues Tyr567-Ser993) was expressed in Sf9 insect cells transfected with the baculovirus containing pBac-PAK8-GST-FLT3-KD plasmid. FLT3^WT^ Kinase-Glo assay was carried out in 96-well plates at 30°C for 4.0 h with tested compounds at a final volume of 50 μL including the following components: 75 ng GST-FLT3-KD^WT^ proteins, 25 mM HEPES, pH 7.4, 4.0 mM MnCl_2_, 10 mM MgCl_2_, 2.0 mM DTT, 0.02% Triton X-100, 0.1 mgmL^-1^ bovine serum albumin, 25 μM peptide substrate (GGMEDIYFEFMGGKKK), 0.5 mM Na_3_VO_4_, and 1.0 μM ATP.

### AURKA inhibition assay

AURKA assay was conducted as previously described [18]. Briefly, the recombinant GST-AURKA (residues Ser123-Ser401) containing the kinase domain was expressed in Sf9 insect cells. The kinase assay was carried out in 96-well plates with tested compounds in a final volume of 50 μL at 37°C for 90 min with the following components: 50 mM Tris-HCl pH 7.4, 10 mM NaCl, 10 mM MgCl_2_, 0.01% BSA, 5.0 μM ATP, 1 mM DTT, 15 μM tetra(-LRRASLG) peptide, and 150 ng recombinant AURKA.

### AURKB inhibition assay

AURKB assay was conducted as previously described [49]. Briefly, the recombinant full length His-tagged Aurora-B (residues M1∼A344) was purchased from Invitrogen (Catalog number: PV6130). The kinase assay was carried out in 96-well plates with the tested compound in reaction solution (50 mM Tris-HCl pH 7.4, 10 mM NaCl, 10 mM MgCl_2_, 0.01% BSA, 5 mM ATP, 1 mM DTT and 15 mM tetra(LRRASLG) peptide, and 40 ng recombinant Aurora-B) at 30°C for 180 min.

Following incubation, 50 μL Kinase-Glo Plus Reagent (Promega, Madison, WI, USA) was added, and the mixture was incubated at 25°C for 20 min. A 70 μL aliquot of each reaction mixture was transferred to a black microliter plate, and luminescence was measured on a Wallac Vector 1420 multilabel counter (PerkinElmer, Shelton, CT, USA).

### Cell lines and MTS cell viability assay

The MTS cell viability assay was conducted as previously described [48]. The MOLM-13 human leukemia cell line was obtained from the German Resource Centre for Biological Material (DSMZ, Braunschweig, Germany); the MV4-11 cell line was purchased from the American Type Culture Collection (ATCC, Manassas, VA, USA). Both the leukemia cell lines were maintained in RPMI 1640 medium supplemented with 10% fetal bovine serum (FBS), 10 UmL^-1^ penicillin, and 10 gmL^-1^ streptomycin at 37°C and 5% CO_2_. The Detroit-551, COLO205, and Mia-PaCa2 cell lines were purchased from the American Type Culture Collection (ATCC, Manassas, VA, USA) and cultured according to instructions of ATCC. To determine cell viability after drug treatment, assay for MOLM-13 and MV4-11 was performed by seeding 10000 cells per well in a 96-well culture plate. Detroit-551, COLO205, and Mia-PaCa2 were seeded at density of 2500, 4000, and 3750 cells per well, respectively. After 16 h, cells were then treated with vehicle or various concentrations of compound in medium for 72 h. Viable cells were quantified using the MTS method (Promega, Madison, WI, USA) according to the manufacturer's recommended protocol. Results were determined by measuring the absorbance at 490 nm using a plate reader (Victor2; PerkinElmer, Shelton, CT, USA). The IC_50_ value was defined as the amount of compound that caused a 50% reduction in cell viability in comparison with DMSO-treated (vehicle) control and was calculated using Prism version 4 software (GraphPad, San Diego, CA, USA).

### Western blot analysis

Western blot analysis was conducted as previously described [13]. Primary antibodies against phospho-FLT3 (Tyr591) (#3461) and phospho-AURKA (Thr288) (#3079) were purchased from Cell Signaling Technology. The anti-FLT3 antibody (sc-480) and anti-AURKA antibody (07–648) were purchased from Santa Cruz Biotechnology and Upstate, respectively. The anti-β-actin rabbit polyclonal antibody (GTX110564) was purchased from GeneTex (CA, USA). The secondary antibodies horseradish peroxidase (HRP)-linked goat anti-rabbit IgG (111-035-003) were purchased from Jackson Immuno (West Grove, PA, USA). MV4-11 cells were incubated with compound BPR1K871 for 2.0 h at the indicated concentrations. Cell lysates were prepared and analyzed by immunoblotting. For AURKA analysis, the cell lysates were obtained from MV4-11 cells incubated for 16 h with 40 ngmL^-1^ nocodazole, followed by drug treatment for 2.0 h at the indicated concentrations.

### *In vivo* pharmacokinetics evaluation of BPR1K871 in rats

This study was approved by Institutional Animal Care and Use Committee of National Health Research Institutes. A solution of test compound (10 mg/mL) was prepared by dissolving appropriate amount of compound in a mixture of DMA/PEG400 (20/80, v/v). Male Sprague-Dawley rats, weighing 250–350 g each (8–10 weeks old), were obtained from BioLASCO, Ilan, Taiwan. A single 5.0 mg/kg and 20 mg/kg dose of compound BPR1K871 was separately administered to groups of 3 rats each intravenously (iv) and oral gavage (po), respectively. The volume of dosing solution administered was adjusted according to the body weight recorded before dose administration. At 0 (prior to dosing), 2, 5, 15, and 30 min and at 1, 2, 4, 6, 8, and 24 h after dosing, a blood sample (∼150 μL) was collected from each animal via the jugular-vein cannula and stored in ice (0–4°C). Plasma was separated from the blood by centrifugation (14000 g for 15 min at 4°C in a Beckman model AllegraTM 6R centrifuge) and stored in a freezer (–60°C). All samples were analyzed for the test compound by LC-MS/MS (ABI3000). Data were acquired via multiple reactions monitoring. Plasma concentration data were analyzed with standard noncompartmental method with WinNonLin software program (version 1.1, Pharsight Corporation, CA).

### *In vivo* evaluation of BPR1K871 in subcutaneously xenograft tumor models

Eight week old, male nude mice (*Nu-Fox1*^nu^) were purchased from BioLASCO (Taipei, Taiwan, R.O.C.). Nude mice (*n* = 5–7 per group) were inoculated subcutaneously with MOLM-13 (1 × 10^6^ per flank) or MV4-11 cells (5 × 10^6^ per flank). When the tumor size reached 500 mm^3^, animals were grouped and treated with BPR1K871 at various doses in a 2-week treatment period as indicated. Animals were treated with BPR1K871 (1, 3 and 10 mg/kg, iv) or vehicle as control at once daily for 5 days per week for 2 weeks. Nude mice (*n* = 8–9 per group) were inoculated subcutaneously with Mia-PaCa2 (1 × 10^6^ per flank) or COLO205 cells (1 × 10^6^ per flank). When the tumor size reached ∼200 mm^3^, animals were grouped and treated with BPR1K871 at 20 mg/kg dose in a 2-week treatment period as indicated. All human cancer cells were detected as free of *Mycoplasma spp* before they were injected into animals. Tumor volumes were measured and calculated with the formula length × width^2^/2 after initiation of treatments. Tumor size and animal body weight were measured twice a week after tumor cell inoculation. The significant difference between drug treatment and vehicle control were analyzed using one-way *ANOVA* and Student-Newman-Keuls test. The level of statistical significance was set at *P*<0.05. The uses and experimental procedures in animals were approved by the IACUC (Institutional Animal Care and Use Committee) of the National Health Research Institutes.

### Synthesis of BPR1K871

#### *tert*-Butyl(5-(2-((7-(3-chloropropoxy)quinazolin-4-yl)amino)ethyl)thiazol-2-yl)carbamate (16)

The title compound was synthesized by following the standard procedure A and using the reactants/reagents 7-(3-hydroxypropoxy)quinazolin-4(3*H*)-one [17] (15, 701 g, 3.19 mmol, 1.0 equiv), thionyl chloride (7.5 mL), DMF (0.50 mL), *tert*-butyl (5-(2-aminoethyl)thiazol-2-yl)carbamate (774 mg, 3.18 mmol, 1.0 equiv), triethylamine (644 mg, 6.36 mmol, 2.0 equiv), and EtOH (12 mL). The residue was purified by the use of silica gel column chromatography (MeOH/CH_2_Cl_2_, 1:30 to 1:20, as the eluent) to give 16 (861 mg, 1.86 mmol) in 58% yield as brown solid. ^1^H NMR (300 MHz, DMSO-*d*_6_) δ 11.23 (s, 1H), 8.41 (s, 1H), 8.25 (t, *J* = 6.0 Hz, 1H), 8.12 (d, *J* = 9.0 Hz, 1H), 7.19–7.07 (m, 4H), 4.23 (t, *J* = 6.0 Hz, 2H), 3.82 (t, *J* = 6.3 Hz, 2H), 3.78–3.64 (m, 2H), 3.07 (t, *J* = 6.6 Hz, 2H), 2.30–2.15 (m, 2H), 1.45 (s, 9H). LCMS (ESI) m/z: 464 [M + H]^+^.

### 5-(2-((7-(3-Chloropropoxy)quinazolin-4-yl)amino)ethyl)thiazol-2-amine (17)

The title compound was synthesized by following the standard procedure B (method (ii)) and using the reactants/reagents 16 (209 mg, 0.450 mmol, 1.0 equiv), trifluoroacetic acid (0.70 mL), and CH_2_Cl_2_ (2.5 mL). The residue was purified by the use of silica gel column chromatography (MeOH/CH_2_Cl_2_/NH_4_OH, 1:20:0.1 to 1:10:0.1, as the eluent) to give 17 (153 mg, 0.400 mmol) in 98% yield as yellow solid. ^1^H NMR (400 MHz, CD_3_OD) δ 8.55 (s, 1H), 8.12 (d, *J* = 9.2 Hz, 1H), 7.27 (dd, *J* = 9.2, 2.4 Hz, 1H), 7.13 (d, *J* = 2.4 Hz, 1H), 6.73 (s, 1H), 4.31 (t, *J* = 6.0 Hz, 2H), 3.89 (t, *J* = 6.8 Hz, 2H), 3.80 (t, *J* = 6.4 Hz, 2H), 3.07 (t, *J* = 6.8 Hz, 2H), 2.36–2.26 (m, 2H). LCMS (ESI) m/z: 364 [M + H]^+^.

### 1-(3-Chlorophenyl)-3-(5-(2-((7-(3-chloropropoxy)quinazolin-4-yl)amino)ethyl)thiazol-2-yl)urea (18)

The title compound was synthesized by following the standard procedure C and using the reactants/reagents 17 (200 mg, 0.550 mmol, 1.0 equiv), 3-chlorophenyl isocyanate (843 mg, 5.49 mmol, 10 equiv), MeOH (0.50 mL), and CH_2_Cl_2_ (10 mL). The residue was purified by the use of silica gel column chromatography (MeOH/CH_2_Cl_2_, 1:15, as the eluent) to give 18 (178 mg, 0.344 mmol) in 63% yield as white solid. ^1^H NMR (300 MHz, DMSO-*d*_6_) δ 9.14 (bs, 1H), 8.51 (s, 1H), 8.27 (t, *J* = 5.7 Hz, 1H), 8.13 (d, *J* = 9.0 Hz, 1H), 7.69 (s, 1H), 7.35–7.26 (m, 2H), 7.20–7.02 (m, 4H), 4.23 (t, *J* = 6.0 Hz, 2H), 3.82 (t, *J* = 6.9 Hz, 2H), 3.78–3.68 (m, 2H), 3.07 (t, *J* = 6.9 Hz, 2H), 2.28–2.16 (m, 2H). LCMS (ESI) m/z: 517 [M + H]^+^.

### 1-(3-Chlorophenyl)-3-(5-(2-((7-(3-(dimethylamino)propoxy)quinazolin-4-yl)amino)ethyl)thiazol-2-yl)urea (10, BPR1K871)

The title compound was synthesized by following the standard procedure D and using the reactants/reagents 18 (680 mg, 1.31 mmol, 1.0 equiv), dimethylamine (40 wt. % in H_2_O, 3.3 mL, 26 mmol, 20 equiv), potassium iodide (131 mg, 0.790 mmol, 0.6 equiv), and DMF (5.0 mL). After the reaction mixture was stirred for 3.0 h and then worked up, the residue was purified by the use of silica gel column chromatography (MeOH/CH_2_Cl_2_/NH_4_OH, 1:20:0.1 to 1:5:0.1, as the eluent) to give BPR1K871 (250 mg, 0.475 mmol) in 36% yield as white solid. ^1^H NMR (300 MHz, DMSO-*d*_6_) δ 10.58 (bs, 1H), 9.16 (bs, 1H), 8.41 (s, 1H), 8.25 (t, *J* = 5.4 Hz, 1H), 8.11 (d, *J* = 9.3 Hz, 1H), 7.70 (s, 1H), 7.32–7.30 (m, 2H), 7.14–7.04 (m, 4H), 4.13 (t, *J* = 6.3 Hz, 2H), 3.76–3.70 (m, 2H), 3.07 (t, *J* = 6.6 Hz, 2H), 2.38 (t, *J* = 7.2 Hz, 2H), 2.16 (s, 6H), 1.92–1.87 (m, 2H). LCMS (ESI) m/z: 526 [M + H]^+^.

## SUPPLEMENTARY MATERIALS FIGURES AND TABLES





## Abbreviations

AML: acute myeloid leukemia
AURKA: Aurora kinase A
AURKB: Aurora kinase B
CHK2: checkpoint kinase 2
CSF1R: colony stimulating factor 1 receptor
COLO205: human colon adenocarcinoma cell line
FLT3: FMS-like receptor tyrosine kinase-3
Mia-PaCa2: human pancreatic cell line
MOLM-13: human leukemia cell line
MV4-11: human leukemia cell line
PDB ID: Protein Data Bank identification number
PDGFR: platelet-derived growth factor receptors
S_N_2: bimolecular nucleophilic substitution
TRKA: tropomyosin receptor kinase A
VEGFR2: vascular endothelial growth factor receptor 2
