# Supplementary material for: Discovery of BPR1K871, a quinazoline based, multi-kinase inhibitor for the treatment of AML and solid tumors: Rational design, synthesis, in vitro and in vivo evaluation
Source: Oncotarget. 2016 Nov 15;7(52):86239–56. doi: 10.18632/oncotarget.13369 (PMC5349910; doi:10.18632/oncotarget.13369)
Supplement: Supplementary file 1 [file oncotarget-07-86239-s001.pdf]

Discovery of BPR1K871, a quinazoline based, multi-kinase inhibitor for the treatment of AML and solid tumors: Rational design, synthesis, in vitro and in vivo evaluation

Supplementary Materials

General Methods

All commercial chemicals and solvents are of reagent grade and were used without further purification unless otherwise stated. All reactions were carried out under dry nitrogen atmosphere and were monitored for completion by TLC using Merck 60 F_{254} silica gel glass backed plates (5 cm × 10 cm); zones were detected visually under UV irradiation (254 nm) or by spraying with phosphomolybdic acid reagent (Aldrich) followed by heating at 80°C. Flash column chromatography was carried out using silica gel (Merck Kieselgel 60, no. 9385, 230−400 mesh ASTM). ^{1}H NMR spectra were obtained with a Varian Mercury-300 or Varian Mercury-400 spectrometers, and the chemical shifts were recorded in parts per million (ppm, δ) and reported relative to TMS or the solvent peak. Low-resolution mass spectra (LRMS) data were measured on an Agilent MSD-1100 ESI-MS/MS system. Purity of the final compounds were determined with an Hitachi 2000 series HPLC system using C-18 column (Agilent ZORBAX Eclipse XDB-C18 5 μm, 4.6 mm × 150 mm) operating at 25°C. Elution was carried out using acetonitrile as mobile phase A and water containing 0.1% formic acid +10 mmol NH_4OAc as mobile phase B. Elution condition: at 0 min, phase A 10% + phase B 90%; at 45 min, phase A 90% + phase B 10%; at 50 min, phase A 10% + phase B 90%; at 60 min, phase A 10% + phase B 90%. The flow-rate of the mobile phase was 0.5 mL/min, and the injection volume of the sample was 5 μL. Peaks were detected at 210 nm. Purity of all the tested compounds were found to be >95% unless otherwise stated.

Synthesis of *tert*-butyl carbamate derivatives 25–29, 16, 36 (Standard procedure A)

A solution containing a quinazolin-4(3*H*)-one (1.0 equiv), thionyl chloride or phosphoryl trichloride (0.99–50 mL), and DMF (0.10–5.0 mL) in toluene (0–2.0 mL) was heated at reflux for 15 h. After the solvent was evaporated, a solution containing the residue, *tert*-butyl (5-(2-aminoethyl)thiazol-2-yl)carbamate [1] (1.0 equiv), and triethylamine (2.0 equiv) in EtOH (5.0–30 mL) was heated at reflux for 15 h. The reaction mixture was concentrated under vacuum, and the residue partitioned between water and CH_2Cl_2. The organic layer was separated, dried over $MgSO_4$, concentrated, and then purified by the use of silica gel column chromatography to give the desired *tert*-butyl carbamate derivative.

Synthesis of amine derivatives 30–34, 17, 37 (Standard procedure B)

Method (i). A solution containing the *tert*-butyl carbamate derivative (1.0 equiv) in 6.0 N $HCl_{(aq)}$ (5.0–20 mL) was stirred at room temperature for 10 h. The mixture was neutralized by the addition of 6.0 N $NaOH_{(aq)}$ and then extracted with CH_2Cl_2. The organics were separated, concentrated under vacuum, and the solid obtained was purified by silica gel column chromatography to get the desired amines.

Method (ii). tert-Butyl carbamate derivative (1.0 equiv) in CH_2Cl_2 (2.0–10 mL) was treated with trifluoroacetic acid (0.70–1.0 mL), and the mixture was stirred at room temperature for 12 h. The reaction mixture was concentrated under vacuum, and then purified by the use of silica gel column chromatography to give the desired amines.

Synthesis of urea derivatives 4–8, 18, 38 (Standard procedure C)

The amine derivative (1.0 equiv) in a mixture of MeOH (0.30–10 mL) and CH_2Cl_2 (2.5–150 mL) was treated with 3-chlorophenyl isocyanate (10 equiv), and the mixture was stirred at room temperature for 16 h. The reaction mixture was then concentrated under reduced pressure and the residue was purified using silica gel column chromatography to give the desired urea.

Introduction of amino solubilizing group by S_N2 reaction for 9, 10 (Standard procedure D)

A solution containing the urea derivative (1.0 equiv), dimethylamine (2.0–20 equiv), and potassium iodide (0.3–3.0 equiv) in DMF (0.50–5.0 mL) was heated at 100 °C for 3.0–15 h. The reaction mixture was concentrated under vacuum, and then purified by the use of silica gel column chromatography to give the desired S_N2 products.

tert-Butyl (5-(2-((6,7-dimethoxyquinazolin-4-yl) amino)ethyl)thiazol-2-yl)carbamate (25)

The title compound was synthesized by following the standard procedure A and using the reactants/reagents 6,7-dimethoxy-4(3*H*)-quinazolinone (20, 33 mg, 0.16 mmol, 1.0 equiv), thionyl chloride (0.99 mL), DMF (0.10 mL), *tert*-butyl (5-(2-aminoethyl)thiazol-2-yl)carbamate (39 mg, 0.16 mmol, 1.0 equiv), triethylamine (32 mg, 0.32 mmol, 2.0 equiv), and EtOH (5.0 mL). The residue was purified by the use of silica gel column chromatography (MeOH/CH_2Cl_2, 1:10, as the eluent) to give 25 (28 mg, 0.065 mmol) in 41% yield as yellow solid. 1H NMR (400 MHz, DMSO-*d6*) δ 11.26 (bs, 1H), 8.37 (s, 1H), 8.10 (t, *J* = 5.6 Hz, 1H), 7.58 (s, 1H), 7.12 (s, 1H), 7.10 (s, 1H), 3.89 (s, 3H), 3.88 (s, 3H), 3.71 (td, *J* = 6.8, 5.6 Hz, 2H), 3.07 (t, *J* = 6.8 Hz, 1H), 1.45 (s, 9H). LCMS (ESI) m/z: 432 [M + H]+.

tert-Butyl (5-(2-(quinazolin-4-ylamino)ethyl) thiazol-2-yl)carbamate (26)

The title compound was synthesized following the standard procedure A and using the reactants/reagents 4-hydroxyquinazoline (21, 300 mg, 2.05 mmol, 1.0 equiv), thionyl chloride (3.0 mL), DMF (0.10 mL), *tert*-butyl (5-(2-aminoethyl)thiazol-2-yl)carbamate (500 mg, 2.05 mmol, 1.0 equiv), triethylamine (416 mg, 4.11 mmol, 2.0 equiv), and EtOH (5.0 mL). The residue was purified by the use of silica gel column chromatography (MeOH/CH_2Cl_2/NH_4OH, 1:10:0.1, as the eluent) to give 26 (566 mg, 1.52 mmol) in 74% yield as yellow solid. 1H NMR (400 MHz, DMSO-*d6*) δ 11.25 (bs, 1H), 8.47(s, 1H), 8.42 (t, *J* = 5.6 Hz, 1H), 8.19 (d, *J* = 8.4 Hz, 1H), 7.75 (dd, *J* = 8.4, 7.0 Hz, 1H), 7.67 (d, *J* = 8.0 Hz, 1H), 7.50 (dd, *J* = 8.0, 7.0 Hz, 1H), 7.09 (s, 1H), 3.75–3.70 (m, 1H), 3.08 (t, *J* = 6.4 Hz, 2H), 1.44 (s, 9H). LCMS (ESI) m/z: 372 [M + H]+.

tert-Butyl (5-(2-((6,7-bis(2-methoxyethoxy) quinazolin-4-yl)amino)ethyl)thiazol-2-yl) carbamate (27)

The title compound was synthesized by following the standard procedure A and using the reactants/reagents 6,7-bis(2-methoxyethoxy)quinazolin-4(3*H)*-one [2] (22, 1.99 g, 6.76 mmol, 1.0 equiv), thionyl chloride (50 mL), DMF (2.0 mL), *tert*-butyl (5-(2-aminoethyl)thiazol-2-yl) carbamate (1.65 g, 6.78 mmol, 1.0 equiv), triethylamine (1.37 g, 13.5 mmol, 2.0 equiv), and EtOH (30 mL). The residue was purified by the use of silica gel column chromatography (MeOH/CH_2Cl_2/NH_4OH, 1:10:0.1, as the eluent) to give 27 (3.29 g, 6.33 mmol) in 94% yield as yellow solid. 1H NMR (400 MHz, DMSO-*d6*) δ 11.24 (bs, 1H), 8.35 (s, 1H), 8.08 (t, *J* = 5.2 Hz, 1H), 7.60 (s, 1H), 7.11 (s, 1H), 7.10 (s, 1H), 4.24–4.18 (m, 4H), 3.74–3.67 (m, 6H), 3.34 (s, 3H), 3.33 (s, 3H), 3.04 (t, *J* = 6.8 Hz, 2H), 1.44 (s, 9H). LCMS (ESI) m/z: 520 [M + H]+.

tert-Butyl (5-(2-((6-(2-methoxyethoxy) quinazolin-4-yl)amino)ethyl)thiazol-2-yl) carbamate (28)

The title compound was synthesized by following the standard procedure A and using the reactants/reagents 6-(2-methoxyethoxy)quinazolin-4(3*H*)-one [3] (23, 220 mg, 1.00 mmol, 1.0 equiv), phosphoryl trichloride (3.0 mL), DMF (0.10 mL), *tert*-butyl (5-(2-aminoethyl) thiazol-2-yl)carbamate (245 mg, 1.01 mmol, 1.0 equiv), triethylamine (207 mg, 2.05 mmol, 2.0 equiv), and EtOH (5.0 mL). The residue was purified by the use of silica gel column chromatography (MeOH/CH_2Cl_2, 1:20, as the eluent) to give 28 (126 mg, 0.282 mmol) in 28% yield as yellow solid. 1H NMR (400 MHz, $CDCl_3$) δ 8.60 (s, 1H), 7.79 (d, *J* = 9.2 Hz, 1H), 7.43 (dd, *J* = 9.2, 6.8 Hz, 1H), 7.09 (d, *J* = 6.8 Hz, 1H), 6.92 (s, 1H), 4.23–4.18 (m, 2H), 3.95–3.87 (m, 2H), 3.80–3.74 (m, 2H), 3.46 (s, 3H), 3.16–3.12 (m, 2H), 1.61 (s, 9H). LCMS (ESI) m/z: 446 [M + H]+.

tert-Butyl (5-(2-((7-(2-methoxyethoxy) quinazolin-4-yl)amino)ethyl)thiazol-2-yl) carbamate (29)

The title compound was synthesized by following the standard procedure A and using the reactants/ reagents 7-(2-methoxyethoxy)quinazolin-4(3*H*)-one [4] (24, 150 mg, 0.681 mmol, 1.0 equiv), thionyl chloride (0.99 mL), DMF (0.10 mL), toluene (2.0 mL), *tert*-butyl (5-(2-aminoethyl)thiazol-2-yl)carbamate (166 mg, 0.682 mmol, 1.0 equiv), triethylamine (138 mg, 1.36 mmol, 2.0 equiv), and EtOH (5.0 mL). The residue was purified by the use of silica gel column chromatography (MeOH/CH_2Cl_2/NH_4OH, 1:10:0.1, as the eluent) to give 29 (215 mg, 0.483 mmol) in 71% yield as yellow solid. 1H NMR (400 MHz, DMSO-*d6*) δ 11.24 (bs, 1H), 8.41 (s, 1H), 8.25 (t, *J* = 5.6 Hz, 1H), 8.11 (d, *J* = 9.2 Hz, 1H), 7.17–7.08 (m, 3H), 4.25–4.20 (m, 2H), 3.74–3.66 (m, 4H), 3.32 (s, 3H), 3.07 (t, *J* = 6.8 Hz, 2H), 1.45 (s, 9H). LCMS (ESI) m/z: 446 [M + H]+.

5-(2-((6,7-Dimethoxyquinazolin-4-yl)amino) ethyl)thiazol-2-amine (30)

The title compound was synthesized by following the standard procedure B (method (ii)) and using the reactants/reagents 25 (21 mg, 0.049 mmol, 1.0 equiv), trifluoroacetic acid (0.70 mL), and CH_2Cl_2 (2.0 mL). The residue was purified by the use of silica gel column chromatography (MeOH/CH_2Cl_2/NH_4OH, 1:20:0.1 to 1:10:0.1, as the eluent) to give 30 (15 mg, 0.045 mmol) in 92% yield as white solid. 1H NMR (400 MHz, DMSO-*d6*)

δ 8.35 (s, 1H), 8.06 (t, *J* = 5.6 Hz, 1H), 7.58 (s, 1H), 7.09 (s, 1H), 6.69 (m, 3H), 3.89 (s, 3H), 3.88 (s, 3H), 3.66 (td, *J* = 6.8, 5.6 Hz, 2H), 2.95 (t, *J* = 6.8 Hz, 1H). LCMS (ESI) m/z: 332 [M + H]+.

5-(2-(Quinazolin-4-ylamino)ethyl)thiazol-2-amine (31)

The title compound was synthesized by following the standard procedure B (method (i)) and using the reactants/reagents 26 (200 mg, 0.538 mmol, 1.0 equiv) and 6.0 N $HCl_{(aq)}$ (7.0 mL). The residue was purified by the use of silica gel column chromatography (MeOH/CH_2Cl_2, 1:10, as the eluent) to give 31 (144 mg, 0.531 mmol) in 99% yield as light yellow solid. 1H NMR (300 MHz, DMSO-*d6*) δ 8.46 (s, 1H), 8.21 (t, *J* = 5.4 Hz, 1H), 8.20 (d, *J* = 8.3 Hz, 1H), 7.75 (dd, *J* = 8.1, 7.1 Hz, 1H), 7.67 (d, *J* = 8.1 Hz, 1H), 7.50 (dd, *J* = 8.3, 7.1 Hz, 1H), 6.67 (s, 3H), 3.67 (td, *J* = 6.9, 5.4 Hz, 2H), 2.95 (t, *J* = 6.9 Hz, 2H). LCMS (ESI) m/z: 272 [M + H]+.

5-(2-((6,7-Bis(2-methoxyethoxy)quinazolin-4-yl)amino)ethyl)thiazol-2-amine (32)

The title compound was synthesized by following the standard procedure B (method (i)) and using the reactants/reagents 27 (3.53 g, 6.79 mmol, 1.0 equiv) and 6.0 N $HCl_{(aq)}$ (20 mL). The residue was purified by the use of silica gel column chromatography (MeOH/CH_2Cl_2/NH_4OH, 1:15:0.1, as the eluent) to give 32 (1.65 g, 3.93 mmol) in 58% yield as white solid. 1H NMR (400 MHz, DMSO-*d6*) δ 8.33 (s, 1H), 8.02 (t, *J* = 5.2 Hz, 1H), 7.60 (s, 1H), 7.10 (s, 1H), 6.68 (s, 1H), 6.67 (s, 2H), 4.24–4.18 (m, 4H), 3.75–3.70 (m, 4H), 3.70–3.61 (m, 2H), 3.35 (s, 3H), 3.34 (s, 3H), 2.93 (t, *J* = 6.8 Hz, 2H) . LCMS (ESI) m/z: 420 [M + H]+.

5-(2-((6-(2-Methoxyethoxy)quinazolin-4-yl)amino)ethyl)thiazol-2-amine (33)

The title compound was synthesized by following the standard procedure B (method (ii)) and using the reactants/reagents 28 (130 mg, 0.292 mmol, 1.0 equiv), trifluoroacetic acid (1.0 mL), and CH_2Cl_2 (2.0 mL). The residue was purified by the use of silica gel column chromatography (MeOH/CH_2Cl_2, 1:20, as the eluent) to give 33 (62 mg, 0.179 mmol) in 62% yield as light yellow solid. 1H NMR (400 MHz, DMSO-*d6*) δ 8.83 (s, 1H), 8.18 (bs, 1H), 7.90 (d, *J* = 2.2 Hz, 1H), 7.77 (d, *J* = 9.2 Hz, 1H), 7.70 (dd, *J* = 9.2, 2.2 Hz, 1H), 6.97 (s, 1H), 4.28–4.21 (m, 2H), 3.90–3.85 (m, 2H), 3.77–3.72 (m, 2H), 3.34 (s, 3H), 3.04 (t, *J* = 6.4, 2H). LCMS (ESI) m/z: 346 [M + H]+.

5-(2-((7-(2-Methoxyethoxy)quinazolin-4-yl)amino)ethyl)thiazol-2-amine (34)

The title compound was synthesized by following the standard procedure B (method (i)) and using the reactants/reagents 29 (215 mg, 0.483 mmol, 1.0 equiv) and 6.0 N $HCl_{(aq)}$ (5.0 mL). The residue was purified by the use of silica gel column chromatography (MeOH/CH_2Cl_2/NH_4OH, 1:15:0.1, as the eluent) to give 34 (141 mg, 0.408 mmol) in 84% yield as white solid. 1H NMR (400 MHz, DMSO-*d6*) δ 8.41 (s, 1H), 8.21 (t, *J* = 5.2 Hz, 1H), 8.11 (d, *J* = 9.2 Hz, 1H), 7.16–7.06 (m, 2H), 6.68 (s, 1H), 6.67 (s, 2H), 4.26–4.20 (m, 2H), 3.73–3.69 (m, 2H), 3.65 (td, *J* = 6.8, 5.2 Hz, 2H), 2.94 (t, *J* = 6.8 Hz, 2H). LCMS (ESI) m/z: 346 [M + H]+.

1-(3-Chlorophenyl)-3-(5-(2-((6,7-dimethoxyquinazolin-4-yl)amino)ethyl)thiazol-2-yl)urea (4)

The title compound was synthesized by following the standard procedure C and using the reactants/reagents 30 (6.6 mg, 0.020 mmol, 1.0 equiv), 3-chlorophenyl isocyanate (31 mg, 0.20 mmol, 10 equiv), MeOH (0.30 mL), and CH_2Cl_2 (2.5 mL). The residue was purified by the use of silica gel column chromatography (MeOH/CH_2Cl_2/NH_4OH, 1:15:0.1, as the eluent) to give 4 (8.3 mg, 0.017 mmol) in 85% yield as yellow solid. 1H NMR (400 MHz, DMSO-*d6*) δ 10.61 (bs, 1H), 9.20 (bs, 1H), 8.39 (s, 1H), 8.15 (bs, 1H), 7.71 (s, 1H), 7.60 (s, 1H), 7.32–7.31 (m, 2H), 7.15–7.06 (m, 3H), 3.90 (s, 3H), 3.89 (s, 3H), 3.74–3.73 (m, 2H), 3.09 (t, *J* = 6.8 Hz, 2H). LCMS (ESI) m/z: 485 [M + H]+.

1-(3-Chlorophenyl)-3-(5-(2-(quinazolin-4-ylamino)ethyl)thiazol-2-yl)urea (5)

The title compound was synthesized by following the standard procedure C and using the reactants/reagents 31 (100 mg, 0.369 mmol, 1.0 equiv), 3-chlorophenyl isocyanate (568 mg, 3.70 mmol, 10 equiv), MeOH (1.0 mL), and CH_2Cl_2 (20 mL). The residue was purified by the use of silica gel column chromatography (MeOH/CH_2Cl_2/NH_4OH, 1:15:0.1, as the eluent) to give 5 (107 mg, 0.251 mmol) in 68% yield as white solid. 1H NMR (300 MHz, DMSO-*d6*) δ 10.43 (bs, 1H), 9.13 (bs, 1H), 8.48 (s, 1H), 8.43 (t, *J* = 4.8 Hz, 1H), 8.20 (d, *J* = 7.5 Hz, 1H), 7.76 (dd, *J* = 8.4, 6.6 Hz, 1H), 7.69–7.66 (m, 2H), 7.51 (dd, *J* = 8.4, 7.5 Hz, 1H), 7.31–7.30 (m, 2H), 7.13 (s, 1H), 7.08–7.04 (m, 1H), 3.75 (dt, *J* = 6.6, 4.8 Hz, 2H), 3.08 (t, *J* = 6.6 Hz, 2H). LCMS (ESI) m/z: 425 [M + H]+.

1-(5-(2-((6,7-Bis(2-methoxyethoxy)quinazolin-4-yl)amino)ethyl)thiazol-2-yl)-3-(3-chlorophenyl) urea (6)

The title compound was synthesized by following the standard procedure C and using the reactants/reagents 32 (1.71 g, 4.08 mmol, 1.0 equiv), 3-chlorophenyl isocyanate (6.27 g, 40.8 mmol, 10 equiv), MeOH (10 mL), and CH_2Cl_2 (150 mL). The residue was purified by the use of silica gel column chromatography (MeOH/CH_2Cl_2/NH_4OH, 1:10:0.1, as the eluent) to give 6 (1.19 g, 2.08 mmol) in 51% yield as yellow solid. 1H NMR (400 MHz, DMSO-*d6*) δ 10.58 (bs, 1H), 9.20 (bs, 1H), 8.35 (s, 1H), 8.07 (t, *J* = 4.8 Hz, 1H), 7.69 (s, 1H), 7.61 (s, 1H), 7.31 (s, 1H), 7.30 (s, 1H), 7.12 (s, 1H), 7.11 (s, 1H), 7.06–7.04 (m, 1H), 4.24–4.19 (m, 4H), 3.74–3.69 (m, 6H), 3.33 (s, 3H), 3.32 (s, 3H), 3.06 (t, *J* = 5.6 Hz, 2H). LCMS (ESI) m/z: 573 [M + H]+.

1-(3-Chlorophenyl)-3-(5-(2-((6-(2-methoxyethoxy)quinazolin-4-yl)amino)ethyl) thiazol-2-yl)urea (7)

The title compound was synthesized by following the standard procedure C and using the reactants/reagents 33 (62 mg, 0.18 mmol, 1.0 equiv), 3-chlorophenyl isocyanate (275 mg, 1.79 mmol, 10 equiv), MeOH (0.30 mL), and CH_2Cl_2 (2.5 mL). The residue was purified by the use of silica gel column chromatography (MeOH/CH_2Cl_2, 1:20, as the eluent) to give 7 (19 mg, 0.038 mmol) in 21% yield as light yellow solid. 1H NMR (400 MHz, DMSO-*d6*) δ 9.27 (bs, 1H), 8.40 (s, 1H), 8.25 (t, *J* = 5.2 Hz, 1H), 7.70 (s, 1H), 7.67 (d, *J* = 2.6 Hz, 1H), 7.63 (d, *J* = 9.2 Hz, 1H), 7.42 (dd, *J* = 9.2, 2.6 Hz, 1H), 7.34–7.28 (m, 2H), 7.14 (s, 1H), 7.08–7.04 (m, 1H), 4.22–4.19 (m, 2H), 3.79–3.69 (m, 4H), 3.32 (s, 3H), 3.09 (t, *J* = 6.8 Hz, 2H). LCMS (ESI) m/z: 499 [M+H]+.

1-(3-Chlorophenyl)-3-(5-(2-((7-(2-methoxyethoxy)quinazolin-4-yl)amino)ethyl) thiazol-2-yl)urea (8)

The title compound was synthesized by following the standard procedure C and using the reactants/reagents 34 (141 mg, 0.408 mmol, 1.0 equiv), 3-chlorophenyl isocyanate (627 mg, 4.08 mmol, 10 equiv), MeOH (1.0 mL), and CH_2Cl_2 (10 mL). The residue was purified by the use of silica gel column chromatography (MeOH/CH_2Cl_2/NH_4OH, 1:10:0.1, as the eluent) to give 8 (175 mg, 0.351 mmol) in 86% yield as yellow solid. 1H NMR (400 MHz, DMSO-*d6*) δ 9.18 (bs, 1H), 8.42 (s, 1H), 8.26 (t, *J* = 5.2 Hz, 1H), 8.12 (d, *J* = 9.2 Hz, 1H), 7.69 (s, 1H), 7.35–7.28 (m, 2H), 7.16–7.03 (m, 4H), 4.26–4.22 (m, 2H), 3.77–3.68 (m, 4H), 3.32 (s, 3H), 3.07 (t, *J* = 6.8 Hz, 2H). LCMS (ESI) m/z: 499 [M + H]+.

tert-Butyl (5-(2-((7-(2-chloroethoxy)quinazolin-4-yl)amino)ethyl)thiazol-2-yl)carbamate (36)

The title compound was synthesized by following the standard procedure A and using the reactants/reagents 7-(2-hydroxyethoxy)quinazolin-4(3*H*)-one [5] (35, 150 mg, 0.727 mmol, 1.0 equiv), thionyl chloride (1.1 mL), DMF (0.10 mL), toluene (2.0 mL), *tert*-butyl 5-(2-aminoethyl)thiazol-2-ylcarbamate (178 g, 0.730 mmol, 1.0 equiv), triethylamine (147 mg, 1.45 mmol, 2.0 equiv), and EtOH (5.0 mL). The residue was purified by the use of silica gel column chromatography (MeOH/CH_2Cl_2, 1:30 to 1:20, as the eluent) to give 36 (253 mg, 0.562 mmol) in 77% yield as yellow solid. 1H NMR (400 MHz, DMSO-*d6*) δ 11.23 (s, 1H), 8.42 (s, 1H), 8.28 (t, *J* = 5.4 Hz, 1H), 8.13 (d, *J* = 9.2 Hz, 1H), 7.20–7.08 (m, 3H), 4.43–4.39 (m, 2H), 4.02–3.98 (m, 2H), 3.73–3.68 (m, 2H), 3.07 (t, *J* = 6.8 Hz, 2H), 1.45 (s, 9H). LCMS (ESI) m/z: 450 [M + H]+.

5-(2-((7-(2-Chloroethoxy)quinazolin-4-yl)amino) ethyl)thiazol-2-amine (37)

The title compound was synthesized by following the standard procedure B (method (ii)) and using the reactants/reagents 36 (253 mg, 0.562 mmol, 1.0 equiv), trifluoroacetic acid (1.0 mL), and CH_2Cl_2 (10 mL). The residue was purified by the use of silica gel column chromatography (MeOH/CH_2Cl_2/NH_4OH, 1:20:0.1 to 1:10:0.1, as the eluent) to give 37 (143 mg, 0.409 mmol) in 73% yield as yellow solid. 1H NMR (400 MHz, DMSO-*d6*) δ 8.40 (s, 1H), 8.24 (t, *J* = 5.6 Hz, 1H), 8.14 (d, *J* = 9.1 Hz, 1H), 7.16 (dd, *J* = 9.1, 2.7 Hz, 1H), 7.10 (d, *J* = 2.7 Hz, 1H), 6.68 (s, 1H), 6.66 (s, 2H), 4.42–4.38 (m, 2H), 4.02–3.98 (m, 2H), 3.68–3.62 (m, 2H), 2.94 (t, *J* = 7.0 Hz, 2H). LCMS (ESI) m/z: 350 [M + H]+.

1-(5-(2-((7-(2-Chloroethoxy)quinazolin-4-yl) amino)ethyl)1thiazol-2-yl)-3-(3-chlorophenyl) urea (38)

The title compo1und was synthesized by following the standard procedure C and using the reactants/reagents 37 (144 mg, 0.412 mmol, 1.0 equiv), 3-chlorophenyl isocyanate (631 mg, 4.11 mmol, 10 equiv), MeOH (0.50 mL), and CH_2Cl_2 (10 mL). The residue was purified by the use of silica gel column chromatography (MeOH/CH_2Cl_2, 1:15, as the eluent) to give 38 (184 mg, 0.368 mmol) in 89% yield as white solid. 1H NMR (400 MHz, DMSO-*d6*) δ 9.23 (bs, 1H), 8.41 (s, 1H), 8.28 (t, *J* = 5.4 Hz, 1H), 8.13 (d, *J* = 9.2 Hz, 1H), 7.68 (s, 1H), 7.32–7.25 (m, 2H), 7.16–7.00 (m, 4H), 4.42–4.39 (m, 2H), 4.02–3.99 (m, 2H), 3.71 (td, *J* = 6.8, 5.4 Hz, 2H), 3.06 (t, *J* = 6.8 Hz, 2H). LCMS (ESI) m/z: 503 [M + H]+.

1-(3-Chlorophenyl)-3-(5-(2-((7-(2-(dimethylamino)ethoxy)quinazolin-4-yl)amino)ethyl)thiazol-2-yl)urea (9)

The title compound was synthesized by following the standard procedure D and using the reactants/reagents 38 (50 mg, 0.099 mmol, 1.0 equiv), dimethylamine (40 wt. % in H_2O, 0.23 mL, 1.8 mmol, 18 equiv), potassium iodide (10 mg, 0.060 mmol, 0.6 equiv), and DMF (1.0 mL). After the reaction mixture was stirred for 15 h and then worked up, the residue was purified by the use of silica gel column chromatography ($MeOH/CH_2Cl_2/NH_4OH$, 1:5:0.1, as the eluent) to give 9 (32 mg, 0.062 mmol) in 63% yield as white solid. 1H NMR (400 MHz, DMSO-*d6*) δ 9.19 (bs, 1H), 8.42 (s, 1H), 8.26 (t, J = 6.0 Hz, 1H), 8.12 (d, J = 9.2 Hz, 1H), 7.69 (s, 1H), 7.35–7.28 (m, 2H), 7.16–7.04 (m, 4H), 4.19 (t, J = 5.6 Hz, 2H), 3.80–3.70 (m, 2H), 3.07 (t, J = 6.8 Hz, 2H), 2.74–2.66 (m, 2H), 2.26 (s, 6H). LCMS (ESI) m/z: 512 [M + H]+. HPLC purity: 88%.

1-(5-(2-((7-(3-(Dimethylamino)propoxy)quinazolin-4-yl)amino)ethyl)thiazol-2-yl)-3-phenylurea (11)

The intermediate 17 (100 mg, 0.275 mmol, 1.0 equiv) in CH_2Cl_2 (10 mL) was treated with phenyl isocyanate (328 mg, 2.75 mmol, 10 equiv), and the mixture was stirred at room temperature for 16 h. The reaction mixture was then concentrated under reduced pressure and the residue was purified by the use of silica gel column chromatography ($MeOH/CH_2Cl_2/NH_4OH$, 1:20:0.1, as the eluent) to give 39. A solution containing 39 and dimethylamine (40 wt. % in H_2O, 0.23 mL, 1.8 mmol, 6.5 equiv) in DMF (2.0 mL) was heated at 100 °C for 6.0 h. The reaction mixture was concentrated under vacuum, and then purified by the use of silica gel column chromatography ($MeOH/CH_2Cl_2/NH_4OH$, 1:10:0.1, as the eluent) to give 11 (59 mg, 0.12 mmol) in 43% yield as light yellow solid. 1H NMR (400 MHz, DMSO-*d6*) δ 8.93 (bs, 1H), 8.39 (s, 1H), 8.23 (bs, 1H), 8.10 (d, J = 8.8 Hz, 1H), 7.43 (d, J = 8.4 Hz, 1H), 7.28 (t, J = 8.0 Hz, H), 7.12–7.00 (m, 5H), 4.11 (t, J = 6.4 Hz, 2H), 3.78–3.71 (m, 2H), 3.06 (t, J = 7.2 Hz, 2H), 2.36 (t, J = 7.2 Hz, 2H), 2.14 (s, 6H), 1.89–1.75 (m, 2H). LCMS (ESI) m/z: 492 [M + H]+.

1-(5-(2-((7-(3-(Dimethylamino)propoxy)quinazolin-4-yl)amino)ethyl)thiazol-2-yl)-3-(3-methoxyphenyl)urea (12)

The intermediate 17 (100 mg, 0.275 mmol, 1.0 equiv) in 1,4-dioxane (2.5 mL) was treated with triethylamine (73 mg, 0.72 mmol, 2.6 equiv) and phenyl

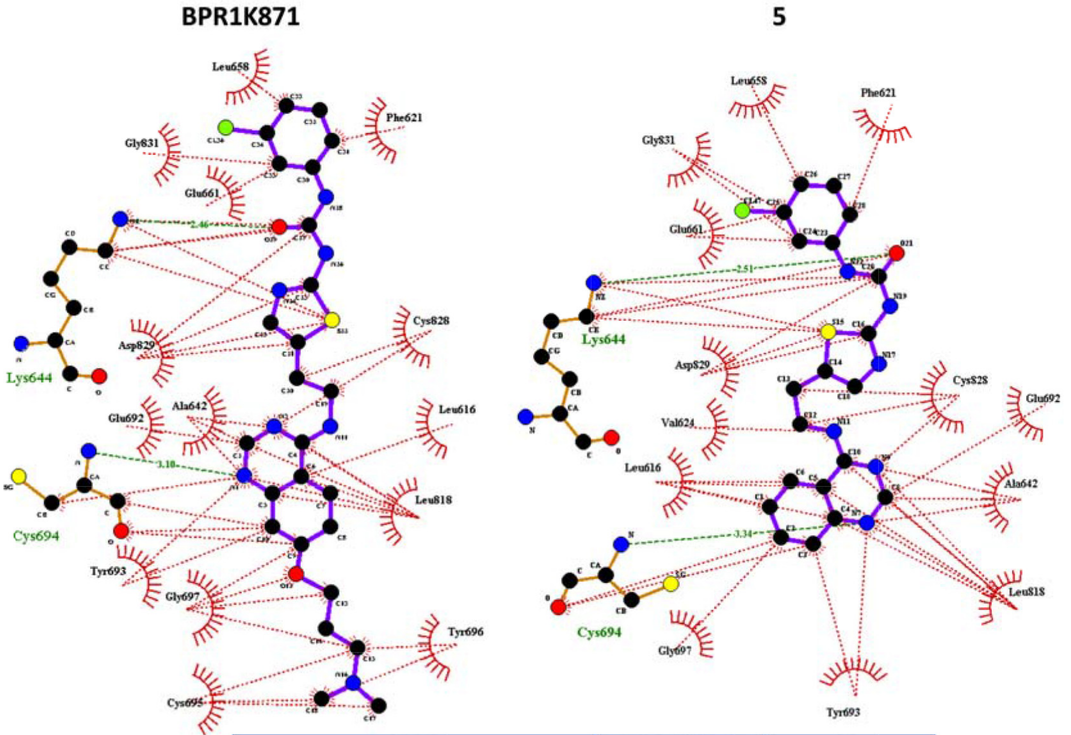

FLT3	BPR1K871	5
IC_{50} (nM)/E_{bind} (kcal mol^{-1})	19/-80.27	1408/-45.75

Supplementary Figure S1: LIGPLOT diagrams and calculated binding energies (E_{bind}) of BPR1K871 and 5 against FLT3 kinase.

(3-methoxyphenyl)carbamate [6] (187 mg, 0.769 mmol, 2.8 equiv), and the mixture was heated at 90 °C for 1.0 h. The reaction mixture was then concentrated under reduced pressure and the residue was purified by the use of silica gel column chromatography (MeOH/CH_2Cl_2/NH_4OH, 1:40:0.1, as the eluent) to give 40. A solution containing 40 and dimethylamine (40 wt. % in H_2O, 0.10 mL, 0.78 mmol, 2.8 equiv) in DMF (1.0 mL) was heated at 100 °C for 6.0 h. The reaction mixture was concentrated under vacuum, and then purified by the use of silica gel column chromatography (MeOH/CH_2Cl_2/NH_4OH, 1:10:0.1, as the eluent) to give 12 (46 mg, 0.088 mmol) in 32% yield as light yellow solid. 1H NMR (400 MHz, $CDCl_3$) δ 8.53 (s, 1H), 7.67 (d, *J* = 8.8 Hz, 1H), 7.22–6.90 (m, 6H), 6.64 (ddd, *J* = 8.2, 2.6, 0.8 Hz, 1H), 4.11 (t, *J* = 6.2 Hz, 2H), 3.84–3.77 (m, 5H), 3.11 (t, *J* = 6.0 Hz, 2H), 2.56 (t, *J* = 7.6 Hz, 2H), 2.31 (s, 6H), 2.07–2.01 (m, 2H). LCMS (ESI) m/z: 522 [M + H]+.

1-(5-Chloro-2-methoxyphenyl)-3-(5-(2-((7-(3-(dimethylamino)propoxy)quinazolin-4-yl)amino)ethyl)thiazol-2-yl)urea (13)

The intermediate 17 (100 mg, 0.275 mmol, 1.0 equiv) in 1,4-dioxane (10 mL) was treated with triethylamine (73 mg, 0.72 mmol, 2.6 equiv) and phenyl (5-chloro-2-methoxyphenyl)carbamate (228 mg, 0.821 mmol, 3.0 equiv), and the mixture was heated at 90 °C for 1.0 h. The reaction mixture was then concentrated under reduced pressure and the residue was purified by the use of silica gel column chromatography (MeOH/CH_2Cl_2/NH_4OH, 1:40:0.1, as the eluent) to give 41. A solution containing 41 and dimethylamine (40 wt. % in H_2O, 0.10 mL, 0.78 mmol, 2.8 equiv) in DMF (1.0 mL) was heated at 100 °C for 6.0 h. The reaction mixture was concentrated under vacuum, and then purified by the use of silica gel column chromatography (MeOH/CH_2Cl_2/NH_4OH, 1:10:0.1, as the eluent) to give 13 (69 mg, 0.12 mmol) in 45% yield as light yellow solid. 1H NMR (400 MHz, DMSO-*d6*) δ 8.87 (bs, 1H), 8.41(s, 1H), 8.25 (t, *J* = 5.6 Hz, 1H), 8.17 (d, *J* = 2.0 Hz, 1H), 8.11 (d, *J* = 9.2 Hz, H), 7.14–7.10 (m, 2H), 7.06–7.01 (m, 3H), 4.12 (t, *J* = 6.6 Hz, 2H), 3.87 (s, 3H), 3.75–3.70 (m, 2H), 3.08 (t, *J* = 7.0 Hz, 2H), 2.38 (t, *J* = 7.2 Hz, 2H), 2.15 (s, 6H), 1.92–1.85 (m, 2H). LCMS (ESI) m/z: 556 [M + H]+.

1-(5-Chloro-2-methylphenyl)-3-(5-(2-((7-(3-(dimethylamino)propoxy)quinazolin-4-yl)amino)ethyl)thiazol-2-yl)urea (14)

The intermediate 17 (100 mg, 0.275 mmol, 1.0 equiv) in 1,4-dioxane (2.5 mL) was treated with triethylamine (73 mg, 0.72 mmol, 2.6 equiv) and phenyl (5-chloro-2-methylphenyl)carbamate (216 mg, 0.825 mmol, 3.0 equiv), and the mixture was heated at 90 °C for 1.0 h. The reaction mixture was then concentrated under reduced pressure and the residue was purified by the use of silica gel column chromatography (MeOH/CH_2Cl_2/NH_4OH, 1:40:0.1, as the eluent) to give 42. A solution containing 42 and dimethylamine (40 wt. % in H_2O, 0.10 mL, 0.78 mmol, 2.8 equiv) in DMF (1.0 mL) was heated at 100 °C for 6.0 h. The reaction mixture was concentrated under vacuum, and then purified by the use of silica gel column chromatography (MeOH/CH_2Cl_2/NH_4OH, 1:10:0.1, as the eluent) to give 14 (15 mg, 0.027 mmol) in 10% yield as light yellow solid. 1H NMR (400 MHz, DMSO-*d6*) δ 8.55 (bs, 1H), 8.41 (s, 1H), 8.26 (t, *J* = 5.6 Hz, 1H), 8.11 (d, *J* = 9.2 Hz, 1H), 8.01 (d, *J* = 2.0 Hz, 1H), 7.22 (d, *J* = 8.0 Hz, 1H), 7.15–7.01 (m, 4H), 4.12 (t, *J* = 6.4 Hz, 2H), 3.75–3.70 (m, 2H), 3.08 (t, *J* = 6.8 Hz, 2H), 2.38 (t, *J* = 7.2 Hz, 2H), 2.21 (s, 3H), 2.16 (s, 6H), 1.93–1.86 (m, 2H). HPLC purity: 87%.

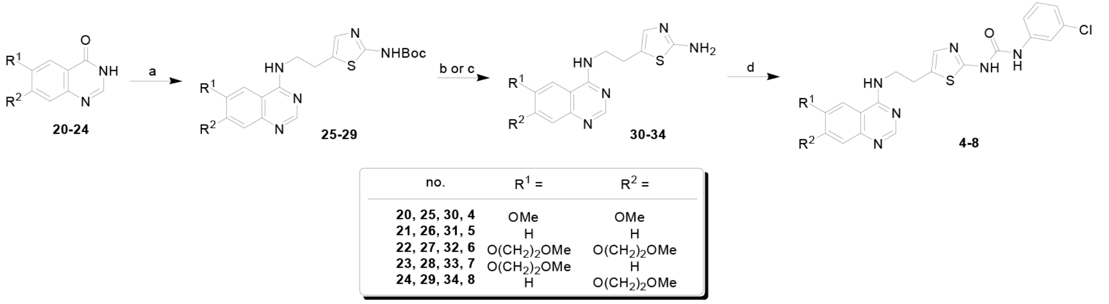

Scheme S1: (A) Method A (i) $SOCl_2$ or $POCl_3$, DMF, reflux, 15 h, (ii) *tert*-butyl (5-(2-aminoethyl)thiazol-2-yl)carbamate, Et_3N, EtOH, reflux, 15 h, 28–94% for two steps; (B) Method B(i), 6.0 N $HCl_{(aq)}$, rt, 10 h, 58–99%; (C) Method B(ii), CF_3CO_2H, CH_2Cl_2, rt, 12 h, 62–92%; (D) Method C, 3-Cl-PhNCO, MeOH, CH_2Cl_2, rt, 16 h, 21–86 %.

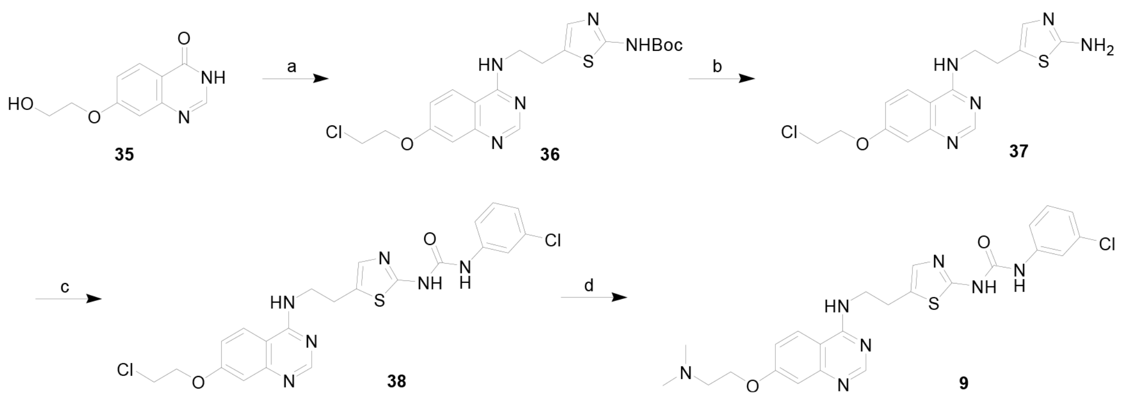

Scheme S2: (A) Method A (i) $SOCl_2$, reflux, 15 h, (ii) *tert*-butyl (5-(2-aminoethyl)thiazol-2-yl)carbamate, Et_3N, EtOH, reflux, 15 h, 77% for two steps; (B) Method B(ii), CF_3CO_2H, CH_2Cl_2, rt, 12 h, 73%; (C) Method C, 3-Cl-PhNCO, MeOH, CH_2Cl_2, rt, 16 h, 89 %; (D) Method D, dimethylamine, KI, DMF, 100 °C, 15 h, 63%.

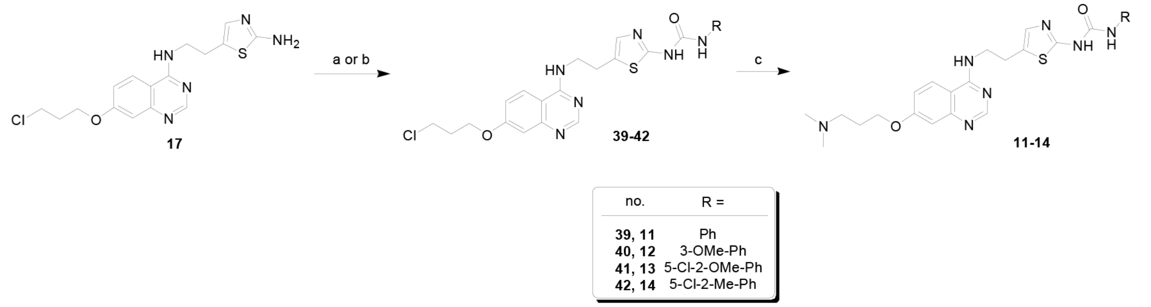

Scheme S3: (A) RNCO, CH_2Cl_2, rt, 16 h; (B) RNHCOOPh, Et_3N, 1,4-dioxane, 90°C, 1.0 h; (C) Me_2NH, DMF, 100°C, 6.0 h, 10–45% for two steps.

Supplementary Table S1: Lipinski's rule of 5 parameters (calculated from http://molinspiration.com/cgi-bin/properties)

Compound number	Mol. Wt.	LogP	H-bond acceptor	H-bond donor
**4**	484.97	4.10	9	3
**5**	424.92	4.47	7	3
**6**	573.08	3.88	11	3
**7**	499.0	4.40	9	3
**8**	499.0	4.40	9	3
**9**	512.04	4.54	9	3
**10**	526.07	4.81	9	3
**11**	491.62	4.15	9	3
**12**	521.65	4.19	10	3
**13**	556.09	4.82	10	3
**14**	540.09	5.21	9	3

Supplementary Table S2: Anti-proliferative activity of BPR1K871 on a panel of in-house leukemia cell lines (Table 3 with SEM data)

Cell Line	Cell type	EC_{50} (nM)[a]					
		BPR1K871	Linifanib	Sorafenib	PKC412	Barasertib	VX-680
MOLM-13	AML-FLT3-ITD (heterozygous)	5 ± 3	38 ± 14	82 ± 37	55 ± 18	42	69 ± 31
MV4–11	AML-FLT3-ITD (homozygous)	4 ± 2	82 ± 17	43 ± 10	38 ± 12	17	71 ± 35
RS4–11	ALL-wt-FLT3 (homozygous)	11 ± 4	9200 ± 2700	9300 ± 1200	400 ± 100	11	nd
U937	AML-FLT3-negative	8050 ± 2616	> 18000[b]	3350 ± 1200	1400 ± 900	> 10000[c]	nd
K562	CML-Bcr-Abl FLT3-negative	2300 ± 800	> 20000[d]	7300 ± 2700	> 20000d	> 10,000[c]	nd

Supplementary Table S3: Pharmacokinetics profile of BPR1K871 in rat

iv			po		
parameter	unit	value	parameter	unit	value
N		3	N		3
dose	mg/kg	5	dose	mg/kg	20
$t_{1/2}$	hr	23.5	$t_{1/2}$	hr	not detectable
clearance	mL/min/kg	60.4	C_{max}	ng/mL	not detectable
Vss	L/kg	34.2	T_{max}	hr	not detectable
AUC (0-∞)	ng/mL*h	1464	AUC (0-∞)	ng/mL*h	not detectable

Supplementary Table S4: Kinase selectivity profiling of BPR1K871 against a 456-kinase panel (containing 395 nonmutant kinases) at a concentration of 1000 nM using the KINOMEScan technology. See Supplementary_Table_S4

Supplementary Table S5: Anti-proliferative activity of BPR1K871 on a panel of in-house non-leukemia cancer cell lines (Table 4 with SEM data)

		EC_{50} (nM)[a]					
**Cell Line**	**Cell type**	**BPR1K871**	**Linifanib**	**Sorafenib**	**PKC412**	**Barasertib**	**VX-680**
HCC827	NSCLC-EGFR[L858R]	257 ± 201	6182 ± 1195	6273 ± 2427	687 ± 285	127 ± 40	nd
H1975	NSCLC-EGFR[LR/TM]	495 ± 231	8853 ± 3040	7010 ± 2485	517 ± 99	390 ± 38	nd
H2228	NSCLC-ALK	212 ± 64	8785	7973	609	340 ± 87	nd
CL-97	Human Lung Cancer	2891 ± 1248	> 20000d	7054 ± 1437	916 ± 54	454	nd
HCT-116	Human Colon Cancer	141 ± 73	8070	8800	482	67	97 ± 33
COLO 205	Human Colon Cancer	34	nd	nd	nd	nd	86
Mia-PaCa2	Human Pancreatic Cancer	94	16369 ± 15	7792 ± 408	995 ± 58	nd	nd
MESSA	Uterine Sarcoma	216	nd	nd	nd	nd	nd
MESSA/DX	Doxorubicin Drug Resisted MESSA	6900	nd	nd	nd	nd	nd
Hep 3B	Human Hepatoma	2471	nd	nd	nd	nd	nd
CL 1–5	Human Lung Adenocarcinoma	1387	nd	nd	nd	nd	nd
MKN-45	Human Gastric Cancer	355	nd	nd	nd	nd	nd

REFERENCES

1. Oslob JD, Romanowski MJ, Allen DA, Baskaran S, Bui M, Elling RA, Flanagan WM, Fung AD, Hanan EJ, Harris S, Heumann SA, Hoch U, Jacobs JW, et al. Discovery of a potent and selective aurora kinase inhibitor. Bioorg Med Chem Lett. 2008; 18:4880–4884.
2. Luth A and Lowe W. Syntheses of 4-(indole-3-yl) quinazolines: a new class of epidermal growth factor receptor tyrosine kinase inhibitors. Eur J Med Chem. 2008; 43:1478–1488.
3. Abraham S, Bhagwat S, Campbell BT, Chao Q, Faraoni R, Holladay MW, Lai AG, Rowbottom MW, Setti E and Sprankle KG. Quinazoline derivatives as Ref kinase modulators and methods of use thereof. WO 2009/117080. 2009.
4. Pandey A, Volkots DL, Seroogy JM, Rose JW, Yu JC, Lambing JL, Hutchaleelaha A, Hollenbach SJ, Abe K, Giese NA, Scarborough RM. Identification of orally active, potent, and selective 4-piperazinylquinazolines as antagonists of the platelet-derived growth factor receptor tyrosine kinase family. J Med Chem. 2002; 45:3772–3793.
5. Heron NM, Pasquet GR, Mortlock AA, Jung FH. Chemical compounds. WO 2004/094410. 2004.
6. Abbasi MA, Sonia A, Rehman AU, Khan KM, Ashraf M, Afzal I, Ambreen N, Shahid M, Abbas M. Synthesis, characterization and biological screening of various *O*-phenyl-*N*-aryl carbamates. J Chem Soc Pak. 2013; 35:385–390.
