# Supplementary material for: Discovery of BPR1K871, a quinazoline based, multi-kinase inhibitor for the treatment of AML and solid tumors: Rational design, synthesis, in vitro and in vivo evaluation
Source: Oncotarget. 2016 Nov 15;7(52):86239–56. doi: 10.18632/oncotarget.13369 (PMC5349910; doi:10.18632/oncotarget.13369)

**Table S4.** Kinase selectivity profiling of **BPR1K871** against a 456-kinase panel (containing 395 nonmutant kinases) at a concentration of 1000 nM using the KINOMEScan technology.


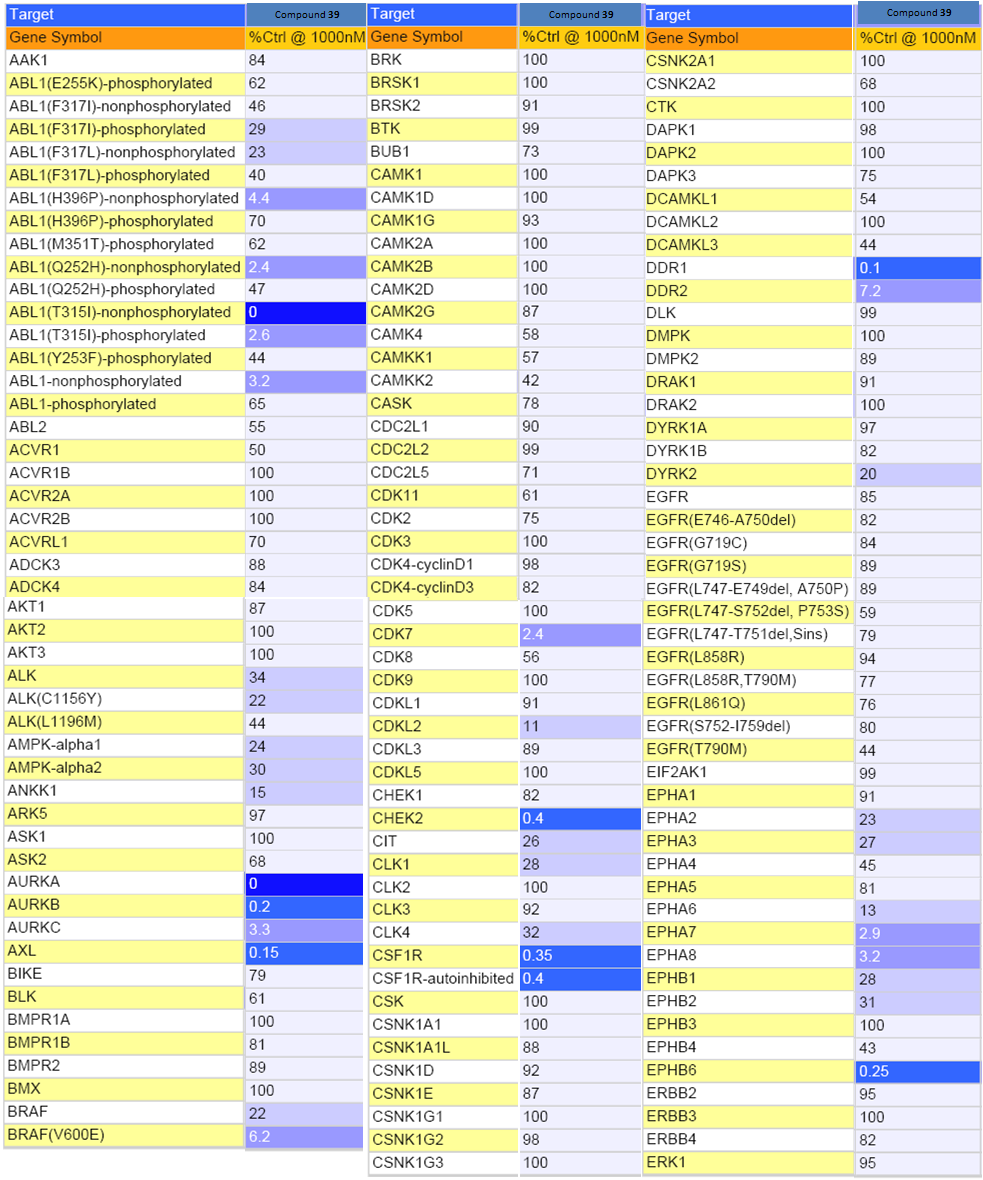


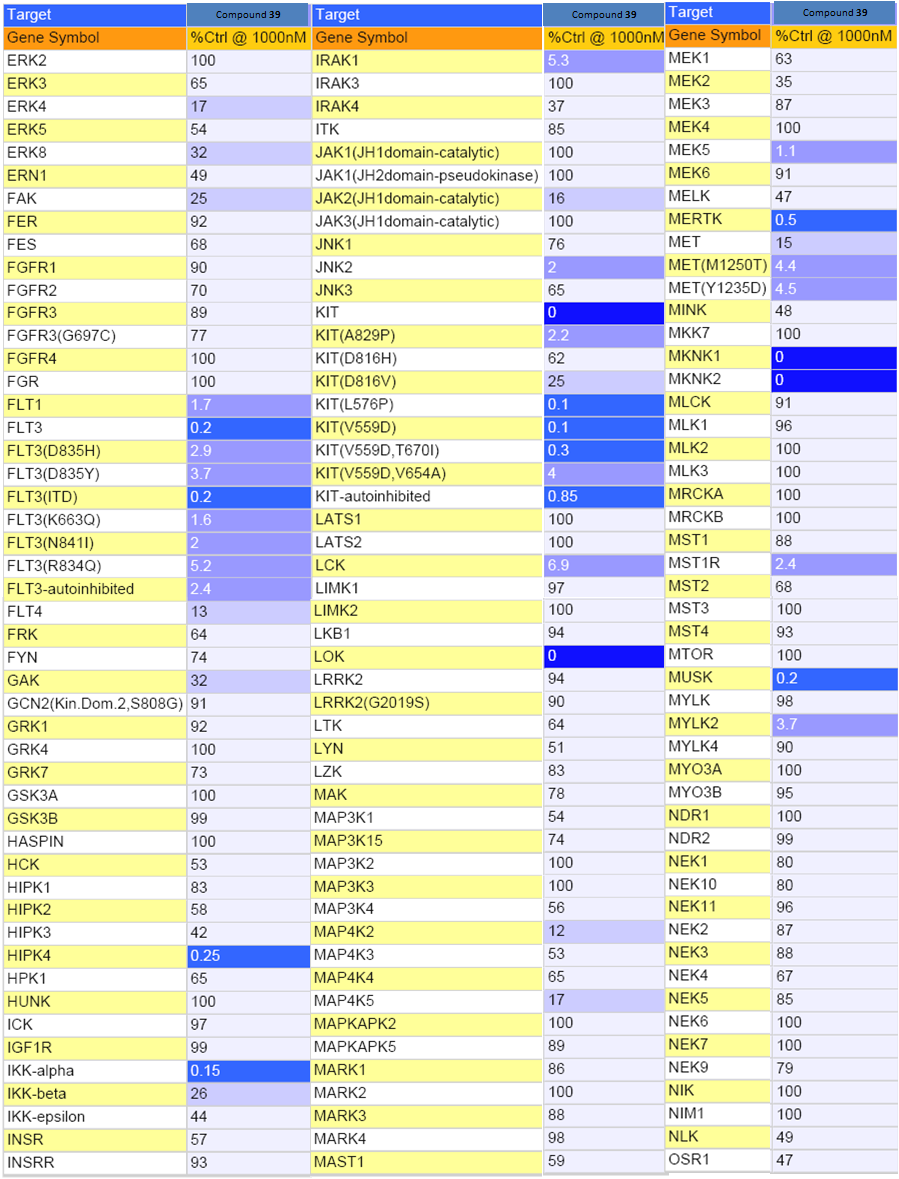


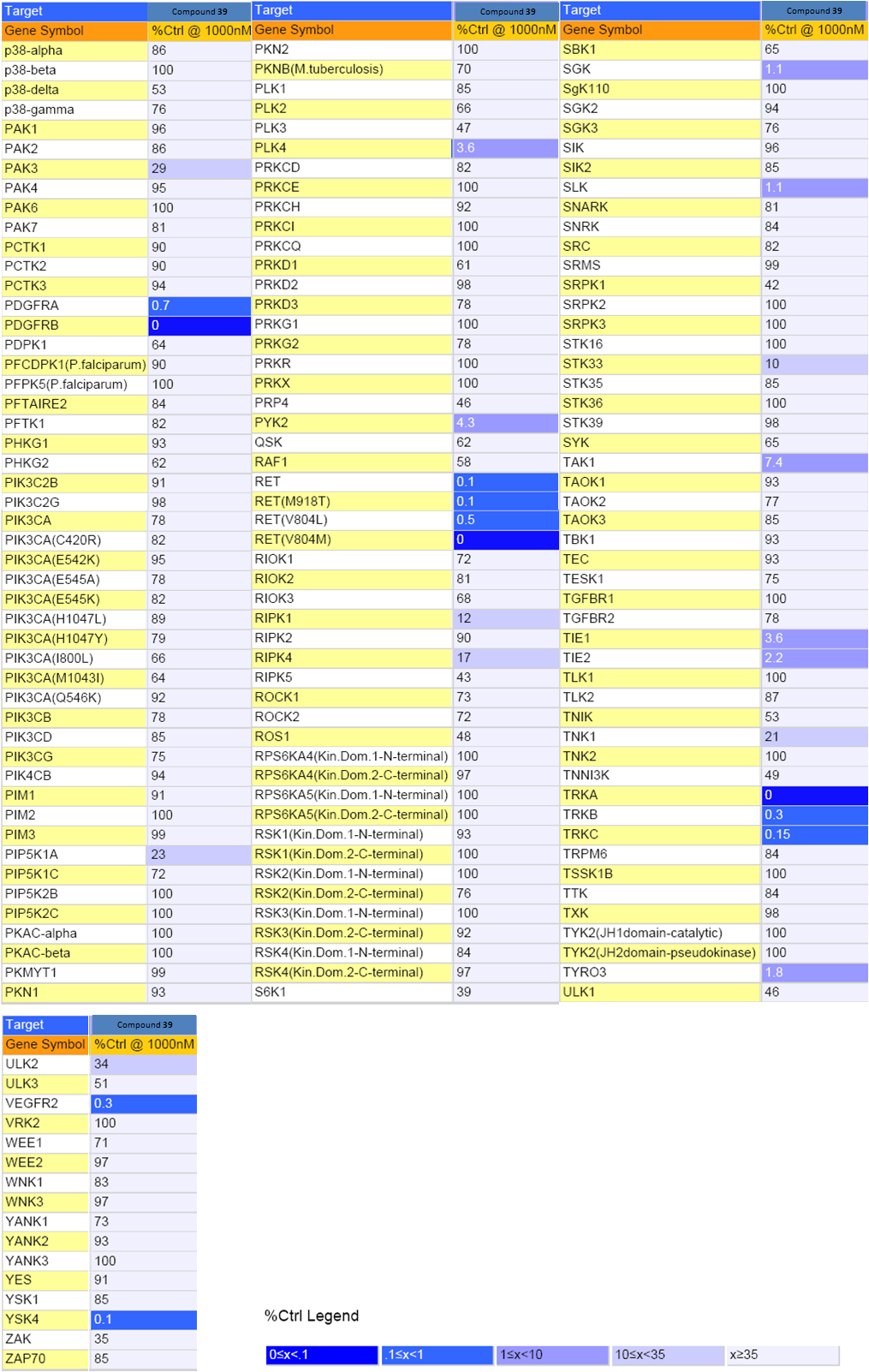

Supplement: Supplementary file 2 [file oncotarget-07-86239-s002.docx]
